# Benchmarking the Elastic Modulus of Conjugated Polymers with Nanoindentation

**DOI:** 10.1021/acs.macromol.4c03081

**Published:** 2025-03-19

**Authors:** Sri Harish Kumar Paleti, Shuichi Haraguchi, Zhiqiang Cao, Mariavittoria Craighero, Joost Kimpel, Zijin Zeng, Przemyslaw Sowinski, Di Zhu, Judith Pons i Tarrés, Youngseok Kim, Qifan Li, Junda Huang, Alexei Kalaboukhov, Besira Mihiretie, Simone Fabiano, Xiaodan Gu, Christian Müller

**Affiliations:** † Department of Chemistry and Chemical Engineering, Chalmers University of Technology, 41296 Göteborg , Sweden; ‡ School of Polymer Science and Engineering, University of Southern Mississippi, Hattiesburg, Mississippi 39406, United States; § Laboratory of Organic Electronics, Department of Science and Technology, Linköping University, 60174Norrköping, Sweden; ∥ Microtechnology and Nanoscience, Chalmers University of Technology, 41296 Göteborg , Sweden; ⊥ Hot Disk AB, Sven Hultins gatan 9A, 41258 Göteborg , Sweden; # Wallenberg Wood Science Center, Department of Chemistry and Chemical Engineering, Chalmers University of Technology, 41296Göteborg, Sweden; ∇ Stellenbosch Institute for Advanced Study, Wallenberg Research Centre at Stellenbosch University, Stellenbosch 7602, South Africa; ▲ Wallenberg Wood Science Center, Department of Science and Technology, Linköping University, 60174 Norrköping, Sweden

## Abstract

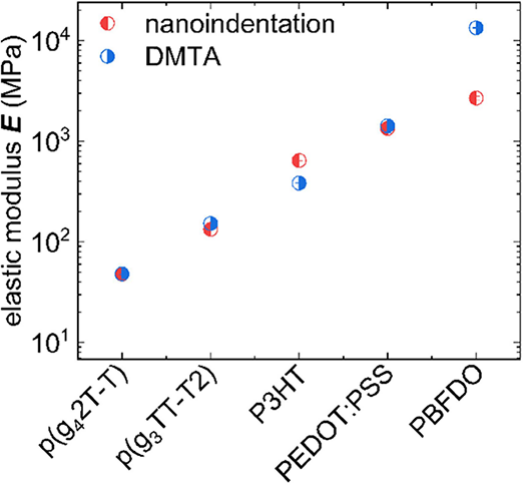

The elastic modulus is a critical parameter for the design of conjugated polymers for wearable electronics and correlates with electrical and thermal transport. Yet, widely different values have been reported for the same material because of the influence of processing and measurement conditions, including the temperature, mode, direction, and time scale of deformation. Thus, results obtained via different methods are usually not considered to be comparable. Here, disparate techniques from nanoindentation to tensile testing of free-standing films or films on water, buckling analysis, dynamic mechanical thermal analysis, oscillatory shear rheometry, and atomic force microscopy are compared. Strikingly, elastic modulus values obtained for the same batch of regioregular poly(3-hexylthiophene) differ by a factor of less than four, which suggests that an approximate comparison is possible. Considering the small amount of material that is typically available, nanoindentation in combination with creep analysis is identified as a reliable method for probing the elastic modulus of films with widely different elastic moduli ranging from less than 0.1 GPa in the case of a polythiophene with oligoether side chains to several GPa for polymers without side chains. Since films can display anisotropic elastic modulus values, it is proposed that nanoindentation is complemented with an in-plane technique such as tensile testing to ensure a full characterization using different modes of deformation.

## Introduction

Conjugated polymers receive considerable interest for the fabrication of wearable electronic devices for energy harvesting, health monitoring, and sensing applications.^1−3^ Detailed knowledge about the mechanical response of each polymer is necessary for selecting suitable materials for specific device geometries and deformation modes. For example, materials with a low stiffness are preferred if the device is meant to accommodate considerable mechanical deformation,^4^ while a high stiffness is more suitable if devices with a rigid configuration are envisaged.^5^

The tensile stiffness *S* depends on the elastic modulus *E* as well as the dimensions of a polymer film, i.e., its cross-sectional area *A* and initial length *L*_0_ according to
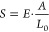
1

Hence, the stiffness of a conjugated polymer film can be adjusted by choosing an appropriate thickness, i.e., a thin film will feature a lower cross-sectional area and thus is less rigid. However, in many cases, optimal device design imposes specific film thickness requirements. Some devices such as thermoelectric generators benefit from micrometer-to-millimeter thick architectures^6^ while others such as solar cells require thin films that are considerably less than one micrometer thick,^7^ which limits the stiffness range for a given material. Instead of varying the film thickness, a material with a suitable elastic modulus can be selected, which is an intrinsic material property that depends on temperature as well as the modes and time scale of deformation.

Another area where the elastic modulus of a conjugated polymer is a useful indicator is electrical and thermal transport, i.e., a stiff conjugated material also tends to be a good electrical and thermal conductor.^8−11^ Moreover, it can be anticipated that for a given porosity and hydrophobicity a soft conjugated material with a low elastic modulus can more easily accommodate the ingression of counterions into polymer films during sequential chemical doping or electrochemical oxidation/reduction cycles (important for, e.g., the switching speed of organic electrochemical transistors, OECTs).^12^ Instead, rigid materials resist diffusion of, e.g., acceptor molecules (important for the thermal stability of organic solar cells).^5^

Evidently, determination of the elastic modulus of a conjugated polymer is important for facilitating an educated selection of materials. A variety of characterization techniques exist that provide information about the elastic modulus. Since the elastic modulus depends on film nanostructure,^13^ temperature and the deformation rate, the results obtained can strongly vary for the same batch of polymer, depending on the chosen deformation mode and experimental conditions. Polymer films can be anisotropic, in which case the measured modulus also strongly depends on the direction (in-plane or out-of-plane) of deformation. Moreover, each measurement technique requires a particular type of sample leading to differences in processing, which can be expected to affect the nanostructure of a polymer film and hence its elastic modulus.

**Figure 1:**
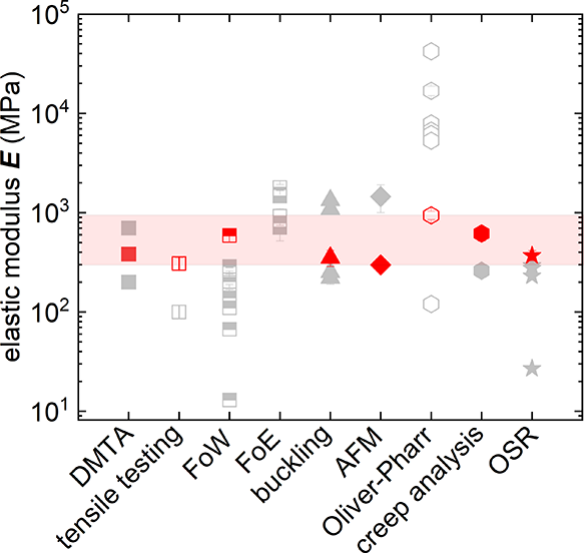
Elastic modulus *E* of regioregular P3HT at room temperature (≈20 °C) measured with dynamic mechanical thermal analysis (DMTA; ■), tensile deformation of free-standing films (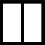), films on water (FoW; ⬒), or film on an elastomer substrate (FoE; ⬓), buckling analysis (▲), PeakForce quantitative nanomechanical mapping (QNM) atomic force microscopy (AFM; ◆), nanoindentation (Oliver-Pharr method ⬡ or creep analysis ⬢), and oscillatory shear rheometry (OSR; ★) from literature (gray symbols; see Table S1 for regioregularity and molecular weight)^11,13,14,16−29^ or measured in this study (red symbols).

For example, regioregular poly(3-hexylthiophene) (P3HT) has been characterized with a variety of techniques ranging from dynamic mechanical thermal analysis (DMTA), tensile deformation (of free-standing films and of films on water or an elastomer substrate), buckling analysis and oscillatory shear rheometry (OSR) to atomic force microscopy (AFM) and nanoindentation. The reported elastic modulus values vary significantly from *E* = 10 MPa to 42 GPa around room temperature (see Figure 1 and Table S1).

Some of this variation may be due to differences in regioregularity and molecular weight, which are known to affect the elastic modulus of P3HT.^9^ In addition, the deformation mode and direction may contribute, especially when comparing methods such as tensile testing and DMTA, which deform a polymer film predominantly in plane (parallel to the surface), with AFM and nanoindentation, in which case the probe tip exerts a force perpendicular to the film surface. It is however important to note that deformation during nanoindentation is not uniaxial but instead comprises a complex pattern comprising both an out-of-plane and an in-plane component. Techniques such as DMTA and OSR involve the application of a small cyclic deformation with a certain frequency, while other techniques such as tensile testing mean that the film experiences a static deformation at a certain rate. Typical samples for tensile testing are free-standing samples that are micrometer to millimeter thick, while tensile deformation of films on water or an elastomer substrate is carried out with submicrometer thin films. The temperature at which the polymers are characterized is another important parameter. The glass transition temperature of regioregular P3HT is located at *T*_g_ = 12–23 °C, measured with differential scanning calorimetry (DSC) or DMTA (loss modulus peak; 1 Hz).^14,15^ As a result, the elastic modulus strongly varies in this temperature range,^9^ meaning that the choice of experimental conditions can significantly affect the apparent value. Since both, material parameters (e.g., regioregularity and molecular weight) and measurement techniques/conditions including the temperature vary between different studies, it is currently not clear to which extent differences in the measured elastic modulus can be assigned to the use of different techniques. We argue that P3HT is a useful reference material because its *T*_g_ is located close to room temperature, which amplifies the impact of various measurement parameters. It can be anticipated that in case of polymers with a much lower or higher *T*_g_ slight variations in measurement parameters have a lesser influence on the obtained elastic modulus.

One technique that tends to yield much higher elastic modulus values for P3HT is nanoindentation (cf. Oliver-Pharr in Figure 1). The technique itself is attractive for the characterization of conjugated polymer films. Samples are typically supported by a substrate and thus solution processing protocols similar to those employed for device fabrication can be used while only small quantities of material are needed (<1 mg). Nanoindentation involves the penetration of a typically micrometer-thick film, for example with a Berkovich tip, which has the shape of a three-sided pyramid (Figure S1). The most widely used method for the analysis of load–displacement curves *P*(*h*) recorded during nanoindentation was introduced by Oliver and Pharr,^30^ which assumes that the deformation of the film occurs within the linear elastic regime. The Oliver-Pharr method follows the polymer deformation at the start of load removal and often overestimates the elastic modulus of polymer films because the material can experience plastic deformation during indentation^31^ and pile up around the area where the film is indented.^32^ As a result, the Oliver-Pharr method tends to yield values of *E* > 1 GPa for P3HT,^32^ reaching up to 42 GPa in one case (see Figure 1 and Table S1).^26^ An alternative method for the analysis of *P*(*h*) curves follows the gradual creep deformation of a polymer film during nanoindentation while maintaining a constant load rate or load, which yields considerably lower values of *E* = 260 MPa.^29^

Here, we investigate to which extent the elastic modulus varies for the same regioregular P3HT batch when measured with nanoindentation as well as a suite of other techniques including tensile testing of free-standing films or films on water (FoW), buckling analysis, dynamic mechanical analysis (DMA), DMTA, OSR and AFM. We chose P3HT as a reference material even though it is not necessarily representative for many high-performance conjugated polymers because it is one of the few materials whose mechanical properties have been characterized with a wide range of techniques (cf. Figure 1) and because its *T*_g_ is located close to room temperature (see above). For the investigated P3HT batch values ranging from *E* = 260 to 938 MPa are obtained, i.e., the measured elastic modulus varies by a factor of less than 4 despite the use of different measurement techniques. No systematic difference is observed between deformation techniques parallel or perpendicular to the surface, suggesting the absence of significant anisotropy. As anticipated, in the case of nanoindentation the Oliver-Pharr method overestimates the elastic modulus while creep analysis yields values that are in better agreement with other techniques.

**Figure 2:**
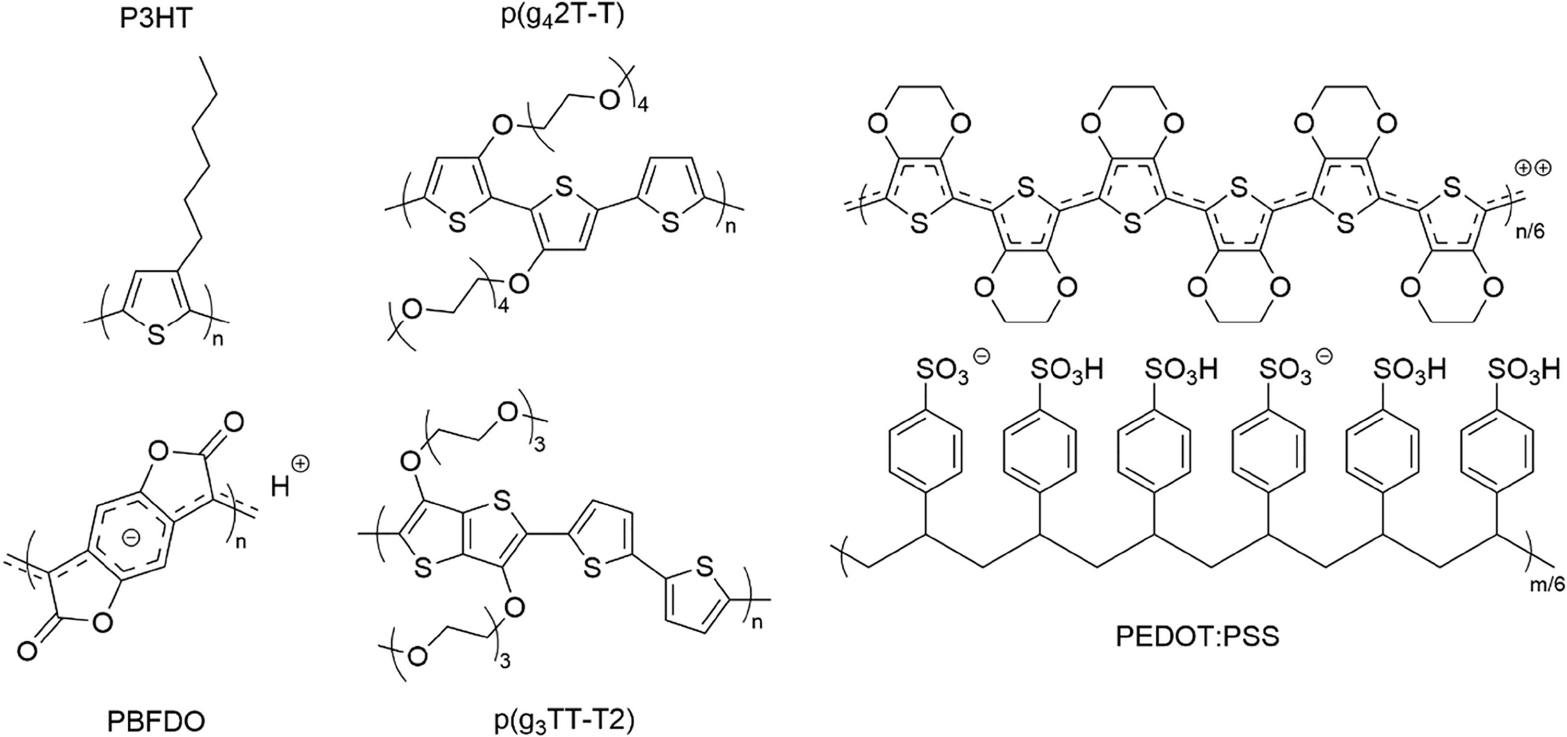
Chemical structures of the investigated materials.

In addition, we carried out a comparison of selected techniques for other types of polymers, i.e., nanoindentation and DMTA/tensile testing. An all-thiophene and a thieno[3,2-*b*]thiophene-based polymer with oligoether side chains, p(g_4_2T-T) and p(g_3_TT-T2) (see Figure 2 for chemical structures), are investigated. These polymers are representative for some of the most widely investigated accumulation mode p-type materials for the fabrication of OECTs^33,34^ and feature an elastic modulus of not more than 100 MPa.^9^ Instead, the depletion mode p-type material poly(3,4-ethylenedioxythiophene):poly(styrenesulfonate) (PEDOT:PSS)^35^ and the recently reported n-type conductor poly(benzodifurandione) (PBFDO)^10^ are reported to be stiff with a modulus exceeding 1 GPa (see Figure 2 for chemical structures).^9,10,36^ For p(g_4_2T-T), p(g_3_TT-T2) and PEDOT:PSS the elastic modulus obtained from nanoindentation, tensile testing and DMTA is in good agreement, suggesting that films are largely isotropic. Evidently, for these materials an approximate comparison of results from different techniques is feasible. For PBFDO films, instead, the mechanical response is anisotropic. A value of about 3 GPa is obtained with nanoindentation, while measurements parallel to the film surface with tensile testing or DMTA yield 9 and 13 GPa, respectively.

## Results and Discussion

In a first set of experiments, we determined the elastic modulus of regioregular P3HT films with a thickness of about 4 μm using nanoindentation. We chose to work with a batch that has a high regioregularity of 98.6% but relatively low number-average molecular weight *M*_n_ = 16 kg mol^–1^, meaning that the polymer is not entangled and features a high tendency to crystallize.^18^ Differential scanning calorimetry revealed a melting temperature *T*_m_ = 230 °C and melting enthalpy of 27 J g^–1^ (Figure S2), which is typical for regioregular P3HT with a similar *M*_n_.^18^

**Figure 3:**
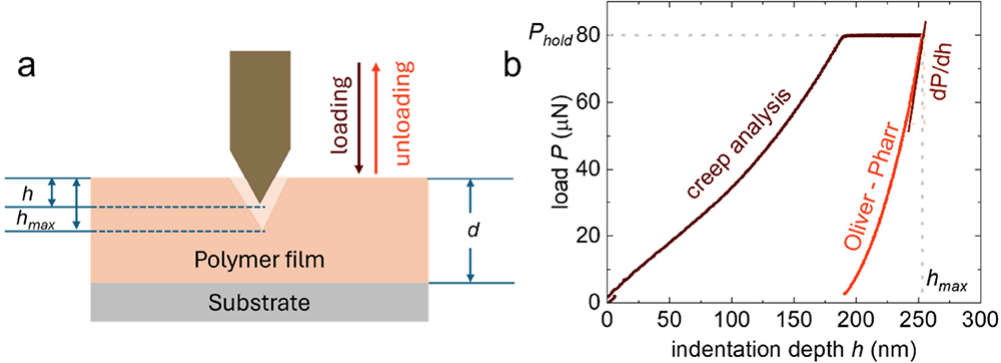
(a) Schematic of a nanoindentation experiment; (b) typical load–displacement curve *P*(*h*) measured for a regioregular P3HT film (thickness *d* ≈ 4 μm; loading rate = 20 μN s^–1^) reaching a maximum applied load *P*_hold_ and maximum indentation depth *h*_max_; creep analysis uses *h*(*t*) recorded during the load/hold segment at *P*_hold_ while the Oliver-Pharr method utilizes the stiffness given by the initial slope of the unloading curve *S* = d*P*/d*h* at *h*_max_.

A nanoindentation measurement entails three steps: (1) a loading segment where the load *P* that the film experiences via the probe tip is gradually increased, (2) a hold segment where a constant load *P*_hold_ is maintained and (3) an unloading segment where the tip is retracted from the film resulting in a decrease of *P* (Figure 3a). The elastic modulus is extracted from the unloading segment or the loading/holding segment of the load–displacement curve *P*(*h*) in case of the Oliver-Pharr method and creep analysis, respectively (Figure 3b).

According to the Oliver-Pharr method the stiffness *S* and reduced elastic modulus *E*_r_ can be calculated from the initial slope of the unloading curve (Figure 3b)^30^:
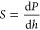
2
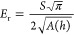
3where *A*(*h*) is the projected area of the indentation tip. Provided the substrate material has a much higher elastic modulus than the polymer film, the elastic modulus *E* of the latter can be obtained from *E*_r_:
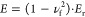
4where ν_f_ is the Poisson’s ratio of the polymer film.

For the here analyzed regioregular P3HT batch a value of *E*_r_ = 1173 ± 104 MPa is obtained (Figure S3). Assuming a Poisson’s ratio of ν_f_ = 0.35, which is a commonly reported value for P3HT (Table S2),^37^ an elastic modulus of *E* = 938 ± 91 MPa is calculated, which is similar to values reported by several previous studies.^13,20,21,25,27,29,38−40^ The Oliver-Pharr method overestimates the elastic modulus because polymer films often undergo plastic deformation upon nanoindentation, which tends to be accompanied by buildup of polymer around the indentation crater.^32^ As a result, the actual contact area with the tip is larger than the assumed projected contact area *A*(*h*), leading to an overestimate of *E*_r_ (cf. eq 3).^32^ Moreover, the presence of the much stiffer substrate can influence the measurement. A typical rule of thumb is that the maximum indentation depth *h*_max_ should not exceed one tenth of the total film thickness *d*.^41^ Since the nanostructure of the top layer of a conjugated polymer film can be different from that of bulk material, it is recommended that the minimum indentation depth is larger than a few tens of nanometers.^42^ Hay and Crawford proposed a model that allows to compensate for interference from the substrate.^43,44^ Using this model, we obtain an elastic modulus of *E* = 845 ± 82 MPa for P3HT, which is similar to the value obtained using the standard Oliver-Pharr method (see Section S1).

Creep analysis instead analyzes gradual deformation of the film during the loading or hold segment. Provided the loading rate is constant, the shear creep compliance *J* as a function of time *t* can be calculated from *P*(*h*) recorded during the loading segment according to^45^
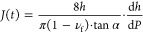
5where the half angle α = 65.27° for a Berkovich tip. d*P*/d*h* can be obtained by taking the first derivative of *P*(*h*), which however strongly fluctuates, complicating a reliable analysis (Figure S4).

Alternatively, *J*(*t*) can be obtained from the hold segment subsequent to rapid loading according to^45^
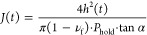
6where *P*_hold_ is a constant load applied by the indentation tip. We opted to use creep analysis in combination with a constant load throughout this manuscript.

**Figure 4:**
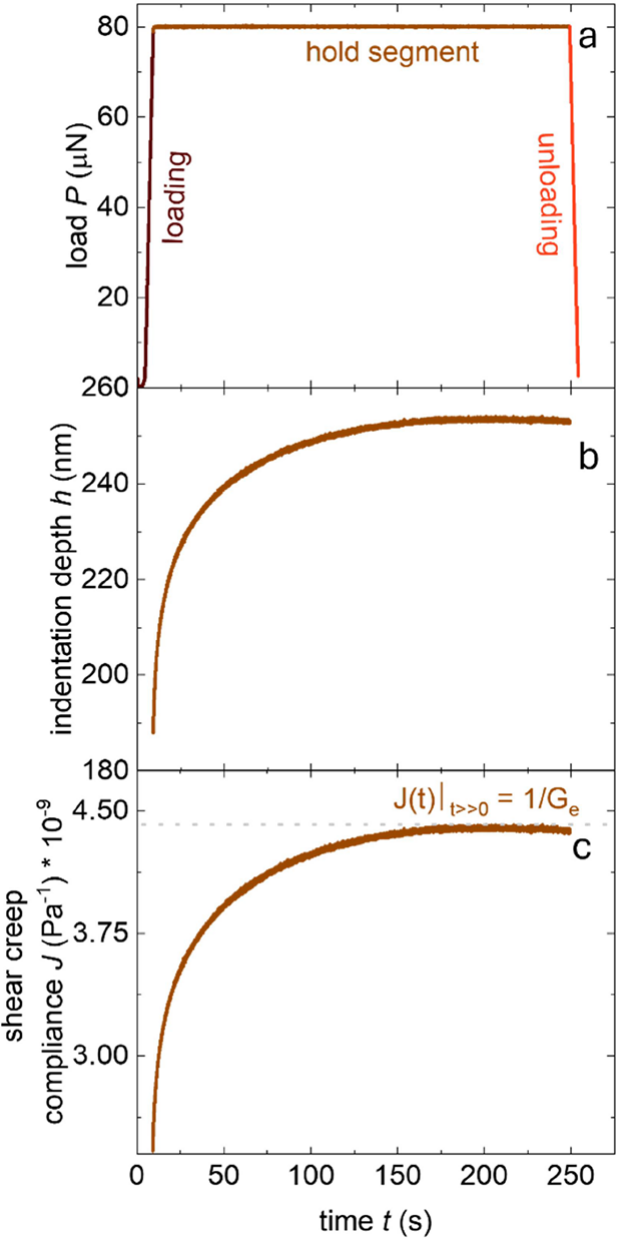
Representative creep analysis of a regioregular P3HT film (thickness *d* ≈ 4 μm; loading rate = 16 μN s^–1^; *P*_hold_ = 80 μN). (a) Load cycle composed of a rapid loading and unloading step as well as a long hold segment during which the load *P*(*t*) = *P*_hold_ is constant with indentation time *t*; (b) displacement curve corresponding to the change in indentation depth *h*(*t*) during the hold segment; and (c) shear creep compliance *J*(*t*) during the hold segment and shear modulus *G*_e_, obtained from *J*(*t*) using eqs 6–8.

For example, an approximately 4 μm thick P3HT film was indented by increasing the load with d*P*/d*t* = 16 μN s^–1^ up to a constant *P*_hold_ = 80 μN (Figure 4a), which resulted in an initial indentation to *h* ≈ 190 nm at the end of the loading segment followed by a gradual increase in *h* to about 250 nm during the hold segment (Figure 4b). Equation 6 was used to determine *J*(*t*) during the hold segment, which first increased and ultimately reached a constant value for *t* ≫ 0 (Figure 4c). The shear and tensile relaxation modulus could then be obtained at longer times according to^46^
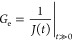
7and
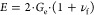
8

We obtain a value of *G*_e_ ≈ 220 MPa (Figure 4c) and *E* ≈ 600 MPa for ν_f_ = 0.35, i.e., the previously reported Poisson’s ratio for regioregular P3HT.^37^

**Figure 5:**
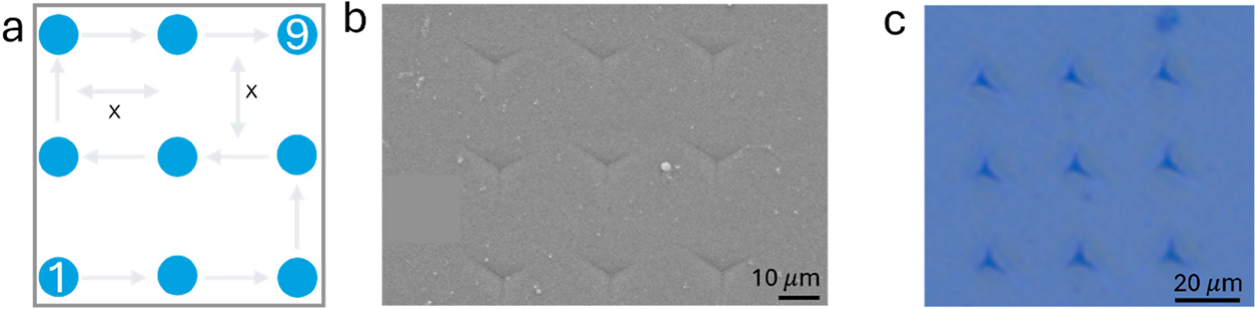
(a) Map of 9 indentation measurements, each yielding one displacement curve (*x* = 15 μm); (b) SEM image and (c) optical micrograph of a P3HT film indented with a constant load of 2000 μN, which was higher than the values used for actual measurements to enlarge the indentation and to make the pile-up around the indentation crater more visible.

**Figure 6:**
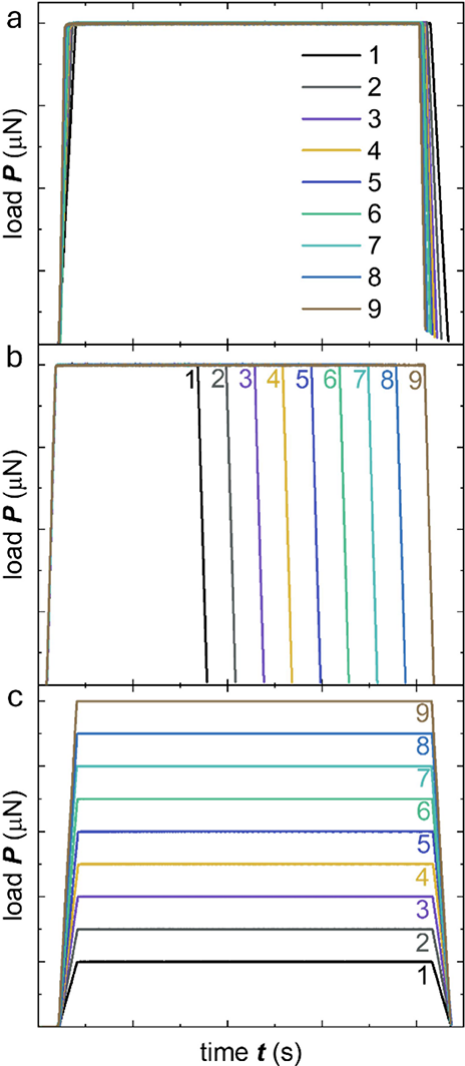
Load applied during a series of 9 creep experiments with (a) the loading/unloading rate varying from d*P*/d*t* = 20 to 854 μN s^–1^ while keeping the same maximum load *P*_hold_ = 80 μN and hold time Δ*t*_hold_ = 75 s; (b) Δ*t*_hold_ varying from 142 to 942 s while keeping the same d*P*/d*t* = 20 μN s^–1^ and *P*_hold_ = 80 μN; and (c) *P*_hold_ varying from 60 to 1500 μN while keeping the same d*P*/d*t* = 20 μN s^–1^ and Δ*t*_hold_ = 75 s.

We investigated to which extent various measurement parameters influence the elastic modulus of P3HT obtained from creep analysis. In particular, we focused on the loading rate d*P*/d*t*, the duration of the hold segment Δ*t*_hold_ and the constant load *P*_hold_ that is maintained during the hold segment. Sequences of nine measurements were carried out (following the pattern shown in Figure 5a) during which one parameter was varied at a time (see Figure 6 for *P*(*t*) and Figure S5 for *h*(*t*) and *J*(*t*)) resulting in a total of 27 measurements.

This approach allowed us to examine potential variations in stiffness across the measured film due to, e.g., variations in the microstructure of the material as well as any potential influence of the measurement parameters. For all measurements, *h*(*t*) and *J*(*t*) gradually increased with time (Figure S5). During most measurements *J*(*t*) approached a constant value, while in case of a high d*P*/d*t* or short Δ*t*_hold_ no steady state had been reached at the end of the hold segment (cf. Figure S5b,e). These measurements were not included in the analysis. Some measurements resulted in a slight decrease in *J*(*t*) toward the end of the hold segment, which we explain with drift of the position of the indentation tip. In these cases, the lowest recorded *J*(*t*) value was used to estimate the elastic modulus.

**Figure 7:**
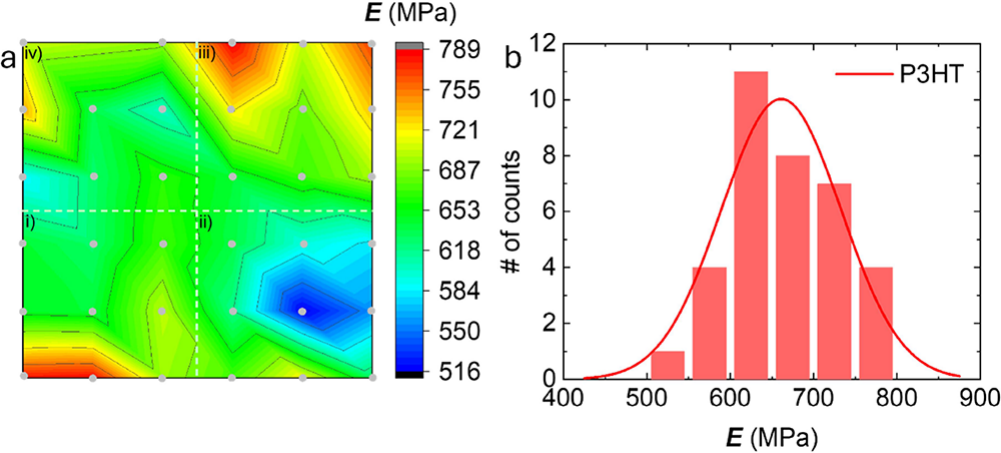
(a) Contour plot of nanoindentation measurements of an approximately 4 μm thick P3HT film as a function of (i) loading/unloading rate d*P*/d*t*, (ii) hold time Δ*t*_hold_, and (iii) maximum load *P*_hold_, and (iv) while maintaining all three parameters constant (d*P*/d*t* = 20 μN s^−1^, Δ*t*_hold_ = 75 s and *P*_hold_ = 80 μN); and (b) frequency of observed *E* values (the solid line is a fit using a Gaussian function).

We observed no systematic variation in elastic modulus with the various measurement parameters d*P*/d*t*, Δ*t*_load_ and *P*_hold_ (Figures 7a i–iii and S6a–c) and therefore treated the maximum of 27 values that we had obtained for *E* as statistically independent. The determined values ranged from 516 to 742 MPa, reflecting the variation of *E* across the film, and overall yielded a mean value and standard deviation of 643 ± 17 MPa. To confirm this result, we carried out nanoindentation in mapping mode at 9 locations across the same film using a constant set of measurement parameters, i.e., d*P*/d*t* = 20 μN s^−1^, Δ*t*_hold_ = 75 s and *P*_hold_ = 80 μN. The elastic modulus varied from 679 to 789 MPa, with a mean value and standard deviation of *E* = 724 ± 36 MPa (Figures 7a iv and S6d). Evidently, mapping of *E* with the same set of measurement parameters results in a similar value as measurements where various parameters are systematically varied. We argue that the observed variation in *E* is due to structural differences across the investigated P3HT film. Finally, the results from all 36 indentation experiments were analyzed together assuming a normal distribution, which yielded a mean value and standard deviation of *E* = 662 ± 74 MPa (Figure 7b).

To benchmark the results from nanoindentation, the elastic modulus of regioregular P3HT was also determined with tensile testing of free-standing films or films on water (FoW), buckling analysis, DMA, DMTA, OSR and AFM. Tensile testing of free-standing and 4 μm thick P3HT films prepared by bar coating was conducted in strain (0.1 mm min^–1^) and force-controlled (5 mN min^–1^) mode, yielding a Young’s modulus of 353 and 307 MPa, respectively (Figure S7a,b). Instead, the tensile elastic modulus of spin coated films measured with the FoW technique had a higher value of 588 ± 9 MPa (Figure S7c), which we tentatively assign to the use of different processing techniques for sample preparation, i.e., blade coating of 4 μm thick films vs spin coating of 115 nm thin films.^47^

For the buckling method 188 nm thick spin coated P3HT films were placed on prestretched polydimethylsiloxane (PDMS) substrates, which were subsequently allowed to relax (Figure S8a). The buckling wavelength λ_b_ was measured at different positions of the same film using optical micrographs of the film surface (Figure S8b). The elastic modulus was calculated according to
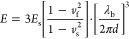
9where *E*_s_ = 1.59 MPa is the elastic modulus of the PDMS substrate (measured with an Instron tensile tester), ν_s_ = 0.5 is the Poisson’s ratio of the PDMS substrate and *d* is the thickness of the P3HT film.

The contraction speed of the PDMS film determines the deformation rate of the P3HT film, which is compressed and hence buckles. We estimate a contraction speed of 24–36 mm min^–1^ by measuring the contraction time and the length of the PDMS film before and after removing the applied force. The buckling method yielded an elastic modulus of *E* = 352 ± 65 MPa, which is comparable with values obtained through tensile testing despite differences in deformation mode and sample geometry (Table 1).

**Table 1:** Elastic Modulus *E* of Regioregular P3HT Measured with Different Methods

method	sample preparation	*T* (°C)	loading/force/strain rate or frequency	*W* × *L* × *d* (mm × mm × μm)^f^	∥/⊥^g^	*E* (MPa)	*n*^h^
static tensile testing (force controlled)	bar coated	18–21	5 mN min^–1^	15 × 4 × 3	∥	307 ± 47	1
static tensile testing (strain controlled)	bar coated	25	0.1 mm min^–1^	4 × 4 × 4	∥	353 ± 2	1
DMA^a^	bar coated	20	0.1–200 Hz	4 × 4 × 77	∥	140–400	1
DMTA^b^	hot pressed	10	1 Hz	7 × 3 × 61	∥	489 ± 3	3
		20				383 ± 2	3
		30				264 ± 5	3
OSR^c^	hot pressed	30	1 Hz	Ø = 8 mm	∥	290 ± 54	3
				*d* = 0.5 mm			
FoW^d^	spin coated	22	4 μm s^–1^	8 × 2 × 0.1	∥	588 ± 9	7
AFM^e^	bar coated	20–22	2000 Hz	25 × 25 × 4	⊥	298 ± 7	3
buckling method	bar coated	18–21	24–36 mm min^–1^	8 × 8 × 0.2	∥/⊥	352 ± 65	3
nanoindentation (Oliver-Pharr)	bar coated	21	20 mN s^–1^	25 × 25 × 4	∥/⊥^i^	938 ± 91	9
nanoindentation (creep analysis)	bar coated	21	20–176 mN s^–1^	25 × 25 × 4	∥/⊥^i^	662 ± 74	36

a Dynamic mechanical analysis (DMA).

b Dynamic mechanical thermal analysis (DMTA).

c Oscillatory shear rheometry (OSR).

d Film-on-water (FOW) tensile testing.

e Atomic force microscopy (AFM).

f Sample dimensions, i.e., width *W*, length *L*, and thickness *d* or diameter Ø and *d*.

g Load direction, i.e., parallel ∥ or perpendicular ⊥ to the surface of the polymer film.

h Number of samples or measurements *n*.

i Note that in case of nanoindentation deformation comprises both an in-plane and out-of-plane component.

OSR of hot pressed P3HT disks was conducted using a constant frequency of 1 Hz while increasing the temperature. The shear storage modulus was *G*′ = 108 ± 20 MPa at 30 °C (Figure S9), yielding a tensile elastic modulus of *E* = 290 ± 54 MPa according to eq 8, again similar to the tensile elastic modulus measured with tensile testing (Table 1). Despite using the same temperature as tensile deformation, *E* is lower in case of OSR compared to tensile testing, confirming that the deformation mode must also be considered.

Quantitative nanomechanical mapping (QNM) atomic force microscopy (AFM) was conducted to investigate the impact of the deformation direction. AFM probes a polymer film perpendicular to the film surface and is more sensitive to the top layer of the film. The recorded force curves were fitted with the Derjagin-Muller-Toropo (DMT) model to extract *E*_r_ according to^48^
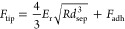
10where *F*_tip_ is the force experienced by the probe tip, *F*_adh_ is the adhesion force, *R* the radius of the probe tip and *d*_sep_ is the separation between sample surface and tip. The elastic modulus was obtained from *E*_r_ using eq 4, yielding a value of *E* = 298 ± 7 MPa (Figure S10). AFM yielded a slightly lower value compared to nanoindentation, which we rationalize with differences in deformation mode. Moreover, AFM is sensitive to the film surface while nanoindentation probes the bulk of the film (cf. Figure 4b; films were indented to a depth of more than 200 nm).

**Figure 8:**
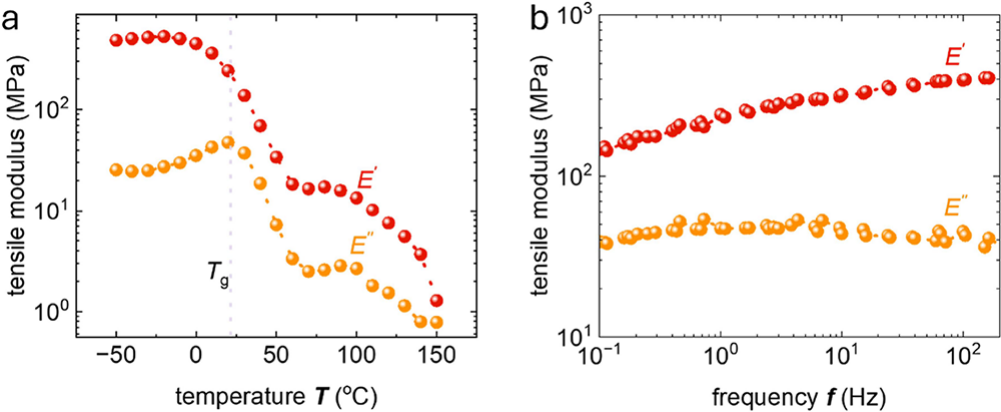
Tensile storage and loss modulus, *E*′ and *E*″, of a 4 μm thick free-standing bar coated P3HT film, measured with a dynamic mechanical analyzer as (a) a function of temperature at 1 Hz (DMTA) and (b) as a function of frequency at 20 °C (DMA); the peak in *E*″ indicates a glass transition temperature *T*_g_ = 21 °C.

Overall, values obtained when probing P3HT films with nanoindentation and AFM span a similar range as tensile testing, buckling and OSR, which probe samples in-plane in tension, compression or shear. Hence, we argue that the investigated P3HT samples do not feature any significant anisotropy in elastic modulus. Differences in measurement temperature and the time scale of deformation can be anticipated to more strongly impact the measured elastic modulus, especially since measurements were carried out close to the *T*_g_ of regioregular P3HT. To probe the temperature and frequency dependence, we also carried out DMTA and DMA in tensile mode using free-standing films. In case of DMTA the temperature was increased from −50 to 150 °C (or −10 to 50 °C) at a constant frequency of 1 Hz (see Figures 8a and S11 for DMTA of bar coated and hot pressed films, respectively), while DMA involved a gradual increase in load frequency from 0.1 to 200 Hz at a constant temperature of *T* = 25 °C (Figure 8b). The peak in the loss modulus at 21 °C corresponds to the *T*_g_ of the polymer, which confirms that small changes in temperature can strongly affect the storage modulus *E*′. For example, DMTA of bar coated films yields a value of *E*′ = 241 MPa at *T* = 20 °C, which decreases to 176 MPa at *T* = 25 °C (Figure 8a). Likewise, *E*′ varies from 140 to 400 MPa at *T* = 20 °C across the studied frequency range of 0.1 to 200 Hz (Figure 8b). Evidently, since the characterization carried out with various techniques was conducted at room temperature, it is likely that even slight changes in experimental conditions can alter the measured elastic modulus. Regardless, the relatively good agreement of values obtained using various techniques (see Table 1) suggests that an approximate comparison of elastic modulus values is feasible in the case of isotropic films.

**Figure 9:**
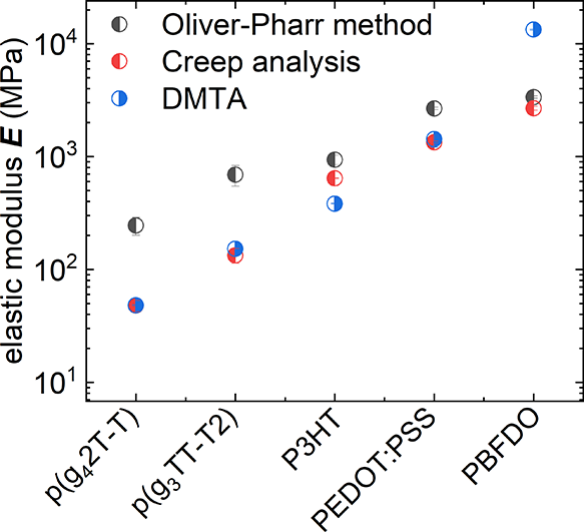
Tensile elastic modulus *E* of various conjugated polymers calculated from the reduced modulus *E*_r_ obtained with the Oliver-Pharr method (black) from the shear modulus *G* obtained with creep analysis assuming a Poisson’s ratio of υ = 0.35 (red) and obtained from DMTA (blue).

In a final set of experiments we used nanoindentation, tensile testing and DMTA to determine the elastic modulus of soft p(g_4_2T-T) and p(g_3_TT-T2), both with oligoether side chains, which tend to result in a *T*_g_ considerably below room temperature,^49,50^ to stiff PEDOT:PSS^51^ and PBFDO.^10,36^ For all polymers we observe that creep analysis yields lower values than the Oliver-Pharr method (Figures 9 and S12–15), with a tendency for bigger differences in case of polymers with a lower elastic modulus, in agreement with previous reports.^18,33,52^ For p(g_4_2T-T), p(g_3_TT-T2) and PEDOT:PSS, values obtained from DMTA and tensile testing (Figures S16 and S17) are in good agreement with values obtained using nanoindentation with creep analysis. These results indicate that measurements of materials, which do not show any thermal transitions close to the measurement temperature (cf. Figure 8; storage modulus of P3HT with a *T*_g_ close to room temperature), are less likely to yield strong differences in elastic modulus. For the two soft polymers p(g_4_2T-T) and p(g_3_TT-T2), nanoindentation indicated an elastic modulus of *E* = 47 ± 5 MPa and 133 ± 20 MPa, while PEDOT:PSS is much stiffer with *E* = 1340 ± 28 MPa (Tables S3–S6). We conclude that the studied p(g_4_2T-T), p(g_3_TT-T2), and PEDOT:PSS films do not display any significant anisotropy in elastic modulus, similar to P3HT films. Here, one could argue that variations in elastic modulus due to the use of different measurement techniques may exactly cancel out due to anisotropy. We would like to point out that neat p(g_4_2T-T) has a very low *T*_g_ = −48 °C and neat films feature no π-stacking,^49^ meaning that any processing induced anisotropy is unlikely to persist at room temperature. Hence, p(g_4_2T-T) films are expected to be isotropic, in agreement with our interpretation of the mechanical measurements.

In case of PBFDO, however, tensile testing and DMTA reveal a much higher elastic modulus of *E* = 8800 ± 200 and 13379 ± 150 MPa, respectively, compared to nanoindentation, which yields *E* = 2680 ± 115 MPa. Evidently, the elastic modulus of the investigated PBFDO films is highly anisotropic, which may be due to preferential in-plane orientation of the polymer backbone due to its rigid nature. We carried out transmission wide-angle X-ray scattering (WAXS) with the surface or the edge of a PBFDO film facing the incoming beam (Figure S18). The recorded WAXS diffraction patterns are comparable to those reported by Sarabia-Riquelme et al. for aligned PBFDO fibers.^10^ Evidently, PBFDO films are characterized by considerable structural anisotropy, consistent with the difference in elastic modulus measured with tensile testing, DMTA and nanoindentation. Hence, we argue that it is advantageous to pair nanoindentation with a second technique that employes a different deformation mode such as tensile testing or DMTA in order to gain insight into potential anisotropy of the elastic modulus of polymer films.

## Conclusions

A wide range of techniques for determining the elastic modulus of conjugated polymer films were compared including nanoindentation, tensile testing, DMTA, OSR and AFM. Investigated materials ranged from soft p(g_4_2T-T) and p(g_3_TT-T2), both with oligoether side chains, to P3HT with alkyl side chains and stiff PEDOT:PSS and PBFDO, both without side chains. Nanoindentation paired with creep analysis is found to be a suitable technique for probing the elastic modulus of conjugated polymer films. Instead, the Oliver-Pharr method results in an overestimate, especially for soft films. In case of p(g_4_2T-T), p(g_3_TT-T2), P3HT and PEDOT:PSS values for the elastic modulus determined by tensile testing and DMTA are in good agreement with creep analysis, suggesting that the investigated films are isotropic. Instead, in case of PBFDO tensile testing and DMTA reveal a higher elastic modulus than values measured with nanoindentation. We conclude that a complete characterization of conjugated polymer films should include techniques that employ different deformation modes, e.g., tensile testing and nanoindentation, in order to gain insight into potential anisotropy of the elastic modulus.

## Experimental Section

### Materials

P3HT with a regioregularity = 98.6% and number-average molecular weight *M*_n_ = 16 kg mol^–1^ (dispersity *Đ*_M_ = 2.9) and an aqueous dispersion of PEDOT:PSS (Clevios PH 1000) were purchased from Ossila Ltd. and Heraeus GmbH, respectively, and used as received. The polymers p(g_4_2T-T) and p(g_3_TT-T2) had an *M*_n_ = 23 and 15 kg mol^–1^ (*Đ*_M_ = 6.5 and 1.8), respectively. The synthesis of PBFDO has been reported elsewhere.^53^ Dodecylbenzenesulfonic acid (DBSA), (3-glycidoxypropyl)trimethoxysilane (GOPS) and ethylene glycol, obtained from Alfa Aesar, Sigma-Aldrich and VWR International, respectively, where used as received. Poly(diallyldimethylammonium chloride) (PDADMAC; weight-average molecular weight *M*_w_ = 400–500 kg mol^–1^) dissolved in water (20 wt %) was obtained from Sigma-Aldrich. Sylgard 184 silicone elastomer base and curing agent were obtained from Sigma-Aldrich. Chlorobenzene (purity 99.8%), chloroform (purity 99.8%), dimethyl sulfoxide (purity 99.9%) and acetonitrile (purity 99.8%) were obtained from Sigma-Aldrich. Acetone (purity 99.8%) and isopropyl alcohol (IPA; purity 99.8%) were obtained from Fischer Scientific. All solvents were degassed for 30 min with argon before ink preparation. Poly(sodium 4-styrenesulfonate) (PSS) and chlorobenzene used for film on water tensile testing were purchased from Sigma-Aldrich and used as received.

### Polymer Solutions

P3HT was dissolved in degassed chlorobenzene at 80 °C (20 g L^–1^) and stirred for 1 h. p(g_4_2T-T) and p(g_3_TT-T2) were dissolved in degassed chloroform at 40 °C (10 g L^–1^) and stirred for 30 min. Ethylene glycol (5 mL L^–1^), GOPS (1 g L^–1^) and DBSA (20 μL L^–1^ vol %) were added to aqueous PEDOT:PSS, followed by stirring at room temperature for 1 h. PBFDO was dispersed in DMSO (5 g L^–1^).

### Nanoindentation

Films of P3HT and p(g_4_2T-T) with a thickness of 4 and 3.7 μm were prepared at room temperature by bar coating 250–300 μL of polymer solution with a K Control Coater from RK Print (wire diameter of 0.08 mm; bar/substrate distance of 1–1.5 mm) at a speed of 10 mm s^–1^ on glass substrates (2.5 cm × 2.5 cm), cleaned by ultrasonication in acetone and then IPA for 8 min, followed by drying under nitrogen. Films of p(g_3_TT-T2) with a thickness of more than 1 μm were drop cast on glass substrates. Films of PEDOT:PSS and PBFDO with a thickness of 48 and 14 μm were prepared by drop casting two times 0.5 mL of dispersion on cleaned glass substrates (2.5 cm × 2.5 cm) at room temperature and 40 °C, respectively. PBFDO films were dried in a vacuum oven at 40 °C for 3 days. Nanoindentation was carried out at room temperature and a humidity of about 31% with a Hysitron TI Premier instrument from Bruker equipped with a Berkovich tip made of diamond with a half angle of 65.27°, calibrated with a reference quartz substrate. Prior to each experiment, the instrument was left in idle condition for 1 h to reach thermal equilibrium. The maximum drift for all experiments was set to 0.02 nm s^–1^, resulting in an error in indentation depth of less than 0.5%. The Oliver-Pharr method and ramp loading experiments were conducted with a loading rate of 20 μN s^–1^; parameters for step loading experiments are detailed in the main text.

### Buckling Method

Samples were prepared by spin coating a thin PDADMAC film onto cleaned glass substrates (500 μL of 5 mL L^–1^ PDADMAC in deionized water, 1000 rpm, 500 rpm s^–1^), followed by spin coating of conjugated polymer films with a thickness of 188 nm (100 μL, 1000 rpm, 500 rpm s^–1^). Then, the PDADMAC layer was dissolved in deionized water and the conjugated polymer films were floated onto prestrained PDMS films (up to 5% elongation) with a thickness of 1.6 mm, which were prepared as described elsewhere.^20^ After conditioning at room temperature overnight, PDMS substrates were allowed to relax, resulting in buckling of the conjugated polymer film. The buckling wavelength and film thickness were determined using an Axio Scope A1 optical microscope from Zeiss and an Alphastep Tencor D-100 profilometer from KLA, respectively.

### Quantitative Nanomechanical Mapping (QNM) Atomic Force Microscopy (AFM)

AFM of 4 μm thick P3HT films (see nanoindentation for sample preparation) was carried out at room temperature with a Dimension ICON instrument from Bruker, equipped with a RTESPA-150–30 cantilever from Bruker with a spring constant of 5 N m^–1^ and a tip radius of *R* = 30 nm. Measurements were carried out using the PeakForce Quantum Nano-Mechanical mapping (QNM) mode. The reduced elastic modulus *E*_r_ was calculated using the DMT model (see eq 10).

### Film-on-Water (FoW) Tensile Testing

Samples were prepared by spin coating a 30 nm thick water-soluble PSS film onto Si wafers,^54^ followed by spin coating of a 115 nm thick P3HT film (20 g L^–1^ in chlorobenzene). The thickness of the films was determined using an Asylum Research Cypher S AFM operating in tapping mode by measuring the step height between the films and the bare silicon wafer. The films were transferred onto a flat silicon substrate for imaging. Following this, P3HT films underwent laser etching to form a dog-bone shape, measuring 2 mm in width and 8 mm in length. Dog-bone-shaped films were floated onto the surface of water, after which two aluminum tensile grips, coated with a thin layer of PDMS (approximately 0.5 mm), were lowered to bond the films through van der Waals forces as described previously.^55^ Tensile testing was done by applying various strains to the film through a motorized linear stage equipped with a digital encoder (Micronix Inc.). Concurrently, the force exerted on the film was monitored using a high-resolution load cell (KYOWA Inc.). Stress–strain curves were derived from force–displacement measurements, with stress calculated as the force divided by the cross-sectional area of the thin film. The strain was determined by measuring the change in sample length relative to its original length.

### Tensile Testing, Dynamic Mechanical Analysis (DMA), and Dynamic Mechanical Thermal Analysis (DMTA)

P3HT coated samples were prepared by first spin coating PDADMAC films on cleaned glass substrates (see buckling method), followed by bar coating conjugated polymer films on top of the PDADMAC layer (see nanoindentation). Then, the PDADMAC layer was dissolved in deionized water and the 4 μm thick free-standing P3HT films were collected from the water surface and dried overnight. Alternatively, P3HT and p(g_3_TT-T2) films were hot pressed (see OSR). Instead, PBFDO and PEDOT:PSS films (see nanoindentation) were peeled from the glass substrate. Tensile testing, DMA and DMTA were performed with a Q800 dynamic mechanical analyzer from TA Instruments. All samples were mounted without any support and a preload force of 1 mN was applied. Tensile testing was performed at 25 °C in controlled force mode at a rate of 5 mN min^–1^ or controlled strain mode at a rate of 0.1 mm min^–1^. DMA was carried out at a temperature of 25 °C and the frequency was changed from 0.1 to 200 Hz. DMTA was carried out at a frequency of 1 Hz while heating from −50 to 150 °C at a rate of 3 °C min^–1^.

### Tensile Testing

A PDMS sample was clamped with pneumatic grips and stretched at a speed of 1 mm min^–1^ with an Instron 5565A instrument.

### Oscillatory Shear Rheometry (OSR)

P3HT samples with a thickness of 0.5 mm were hot pressed with a LabPro200 from Fontijne presses at 200 °C for 2 min. OSR was performed with an MCR 702 instrument from Anton Paar using a disposable aluminum disc with a diameter of 8 mm. Samples were mounted at 200 °C and then cooled to room temperature under controlled axial force conditions (0.5–1.0 N). Measurements were carried out from 0.1 to 100 Hz using a maximum strain of 0.01%, at temperatures ranging from 20 to 140 °C in steps of 10 °C.

### Scanning Electron Microscopy (SEM)

Scanning electron micrographs of nanoindented P3HT films were taken using an Ultra 55 microscope from Zeiss in high vacuum SE 2 mode with an accelerating voltage of 10 kV.

### Optical Microscopy

Optical micrographs were taken in reflection with an Axio Scope A1 microscope from Zeiss.

### Wide Angle X-ray Scattering (WAXS)

A Mat:Nordic instrument from SASXLAB equipped with a Pilatus 300 K detector and a Rigaku 003+ microfocus source (Cu Kα radiation; λ = 1.5406 Å) was used. Transmission mode measurements were performed with a stack of films with the surface or the edge of the films facing the incoming beam.

## Data Availability

The data that support the findings of this study are available online from the Zenodo repository at https://doi.org/10.5281/zenodo.15030674.

## Supporting Information Available

Description of the Hay-Crawford model, Figures S1–S19 and Tables S1–S6 detailing DSC thermograms, nanoindentation measurements, tensile testing experiments, DMTA thermograms and WAXS patterns (PDF)
