# Supplementary material for: Benchmarking the Elastic Modulus of Conjugated Polymers with Nanoindentation
Source: Macromolecules. 2025 Mar 19;58(7):3578–88. doi: 10.1021/acs.macromol.4c03081 (PMC11984310; doi:10.1021/acs.macromol.4c03081)
Supplement: Supplementary file 1 — ma4c03081_si_001.pdf [file ma4c03081_si_001.pdf]

Supporting Information

Benchmarking the elastic modulus of conjugated polymers with nanoindentation

Sri Harish Kumar Paleti,[1]* Shuichi Haraguchi,[1] Zhiqiang Cao,[2] Mariavittoria Craighero,[1] Joost Kimpel,[1] Zijin Zeng,[1,5] Przemyslaw Sowinski,[1] Di Judy Zhu,[1] Judith Pons I Tarrés,[1] Youngseok Kim,[1] Qifan Li,[3] Junda Huang,[3]Alexei Kalaboukhov,[4] Besira Mihiretie,[5] Simone Fabiano,[3] Xiaodan Gu,[2] Christian Müller[1,6,7]*

[1]Department of Chemistry and Chemical Engineering, Chalmers University of Technology, Göteborg 41296, Sweden

*email: paleti@chalmers.se; christian.muller@chalmers.se

[2]School of Polymer Science and Engineering, University of Southern Mississippi, Hattiesburg, MS 39406, USA

[3]Laboratory of Organic Electronics, Department of Science and Technology, Linköping University, Norrköping 60174, Sweden

[4]Microtechnology and Nanoscience, Chalmers University of Technology, Göteborg 41296, Sweden

[5] Hot Disk AB, Sven Hultins gatan 9A, Göteborg 41258, Sweden

[6]Wallenberg Wood Science Center, Department of Chemistry and Chemical Engineering, Chalmers University of Technology, Göteborg, Sweden

[7]Stellenbosch Institute for Advanced Study, Wallenberg Research Centre at Stellenbosch University, Stellenbosch, South Africa

List of supplementary figures

1. Schematic of Berkovich tip
2. Differential scanning calorimetry of P3HT
3. Extracting elastic modulus from $P(h)$ based on Oliver-Pharr method
4. Extracting elastic modulus from $P(h)$ based on creep analysis-constant loading rate mode
5. Indentation depth and shear creep compliance of P3HT as a function of time
6. Contour plots of P3HT's elastic modulus
7. Stress-strain curves of P3HT
8. Buckling analysis of P3HT film
9. OSR of P3HT disks
10. Force curves of P3HT film
11. DMTA thermograms of free-standing P3HT films
12. Statistical representation of p($g_4$2T-T) elastic modulus measured based on creep analysis-constant load mode
13. Statistical representation of p(g_3TT-T2) elastic modulus measured based on creep analysis-constant load mode
14. Statistical representation of PEDOT:PSS elastic modulus measured based on creep analysis-constant load mode
15. Statistical representation of PBFDO elastic modulus measured based on creep analysis-constant load mode
16. DMTA thermograms of free-standing p(g_3TT-T2), p($g_4$2T-T), PEDOT:PSS and PBFDO films
17. Stress-strain curves of free-standing p(g_3TT-T2), p($g_4$2T-T), PEDOT:PSS and PBFDO films
18. WAXS diffractogram of in-plane and out-of-plane measurements on PBFDO film

List of supplementary tables

1. P3HT elastic modulus measured by different techniques as reported in literature
2. Poisson's ratio of P3HT reported in literature
3. Experimental, sample details and elastic modulus of p($g_4$2T-T) measured with different techniques.
4. Experimental, sample details and elastic modulus of p(g_3TT-T2) measured with different techniques.
5. Experimental, sample details and elastic modulus of PEDOT:PSS measured with different techniques.
6. Experimental, sample details and elastic modulus of PBFDO measured with different techniques.

Table S1. Literature values of the elastic modulus E at room temperature (≈20 ºC) of P3HT with a number-average molecular weight M_n, dispersity $Đ_M$ and regioregularity RR; [a]reduced elastic modulus; [b]not specified if M_n or M_w; n.p. = not provided. Shear storage modulus in reference 1 is converted to elastic modulus using equation 8 and ν_f = 0.35.

technique	E(MPa)	M_n(kg mol^{-1})	$Đ_M$ (-)	RR (%)	ref.
OSR	10	45	2.5	59	1
	85	37	1.8	96	1
	102	15	1.2	94	1
	108	28	1.8	98	1
DMTA	200 - 300	91	1.8	93	2
	700	62	1.8	82	3
tensile deformation (free-standing films)	100	29	1.2	96	4
buckling analysis	1330	n.p.	n.p.	n.p.	5
	1090	87	n.p.	98	6
	252	62	1.9	99	7
	220 ± 30	50	2.1	99	8
FoW	135 ± 15	19.6	2.74	≥ 98	9
	287 ± 19	20.3	1.08	98	10
	203 ± 14	15	1.4	95	11
	263 ± 15	40	1.7	95	11
	261 ± 20	63	1.7	> 95	11
	270 ± 12	80	1.6	> 95	11
	287	20.3	1.08	>98	10
	139	20.8	1.23	86	10
	110	13.6	1.42	80	10
	67	12.3	1.44	75	10
	13	9.2	1.33	64	10
FoE	710 ± 190	15	1.4	95	11
	1790 ± 120	40	1.7	95	11
	1460 ± 220	63	1.7	> 95	11
	920 ± 180	80	1.6	> 95	11
	175 ± 25	n.p.	n.p.	n.p.	12
AFM-QNM	1450 ± 450	16	2.9	98.6	13
nanoindentation (Oliver-Pharr)	42200	n.p.	n.p.	n.p.	14
	7900	[b]25	n.p.	n.p.	15
	6700	[b]37	n.p.	n.p.	15
	6200	[b]46	n.p.	n.p.	15
	5300	[b]55	n.p.	n.p.	15
	[a]15000 – 18700	n.p.	n.p.	n.p.	16
nanoindentation (creep analysis)	260	91	1.5	99	17

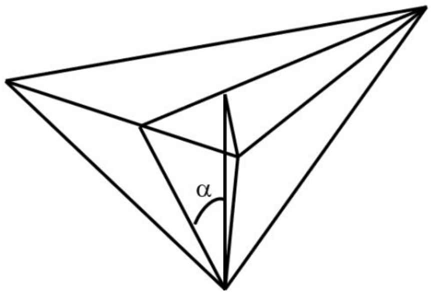

Figure S1. Schematic of a Berkovich tip, α = 65.27°.

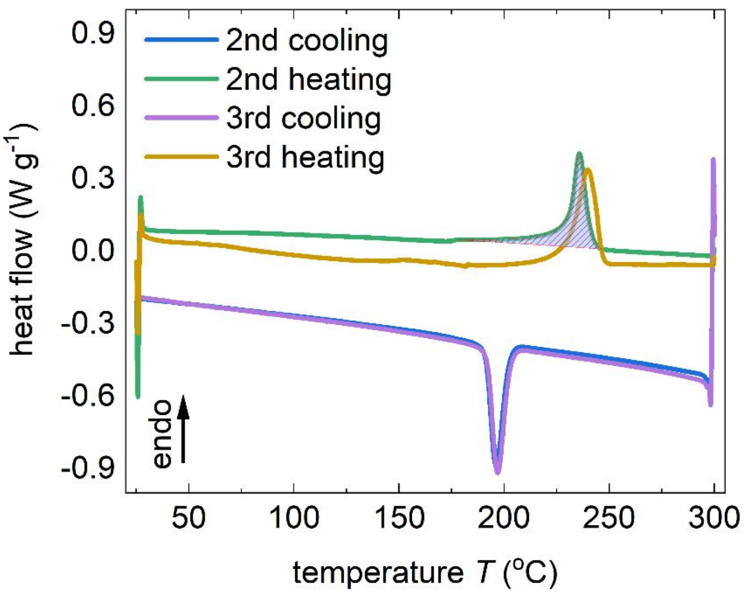

Figure S2. Differential scanning calorimetry (DSC) heating and cooling thermograms of P3HT.

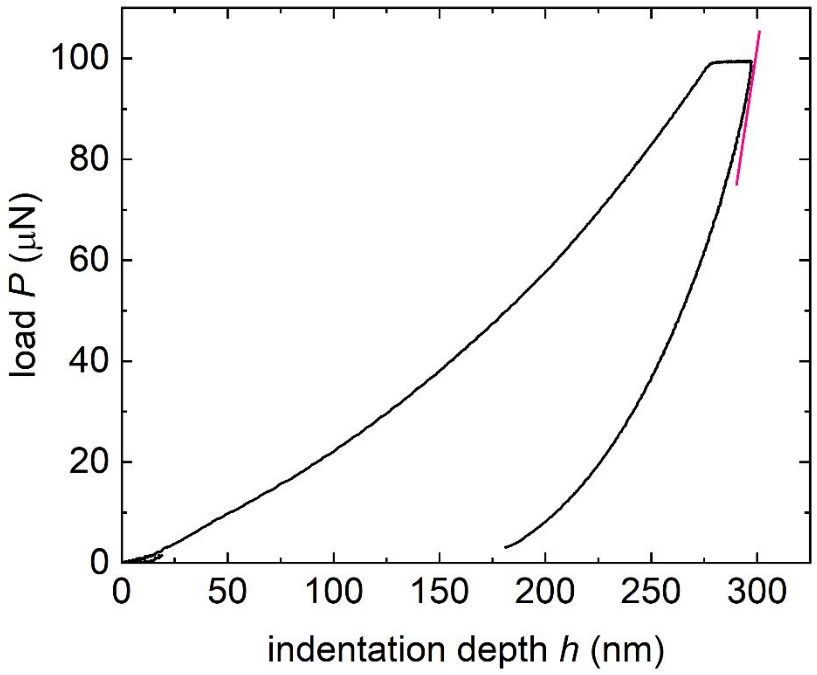

Figure S3. Representative $P(h)$ curve for P3HT to extract elastic modulus based on the Oliver-Pharr method.

Section S1. Hay-Crawford model

Hay-Crawford is an analytical model that allows to account for the response of the substrate when determining the elastic modulus of a polymer film with nanoindentation. The bulk modulus of the film G_f can be calculated according to:[18]

$$G_f = \frac{-B + \sqrt{B^2 - 4AC}}{2A} \quad \text{(S1)}$$

where $A = FI_0$, $B = G_s - (FI_0^2 - I_0 + 1)G_a$, $C = -I_0G_aG_s$. $G_a = E/[2(1 + \upsilon_f)]$ = 347 MPa is the apparent shear modulus of the film (E = 938 MPa is the Young's modulus obtained using the Oliver-Pharr method and υ_f = 0.35), G_s = 26 GPa is the shear modulus of the glass substrate[19], F = 0.0626 is a constant[18] and I_0 is a weighing function that depends on the film thickness, the contact radius of the indentation tip and the Poisson's ratio of the film and substrate. For a film thickness of 4 μm and a contact radius of 544 nm we obtain $I_0 = 0.9$. We obtain A = 0.056, B = 25.948 GPa and C = -8.12 GPa and thus G_f = 313 MPa, which corresponds to $E_f = G_f \cdot 2(1 + \upsilon_f)$ = 845 MPa.

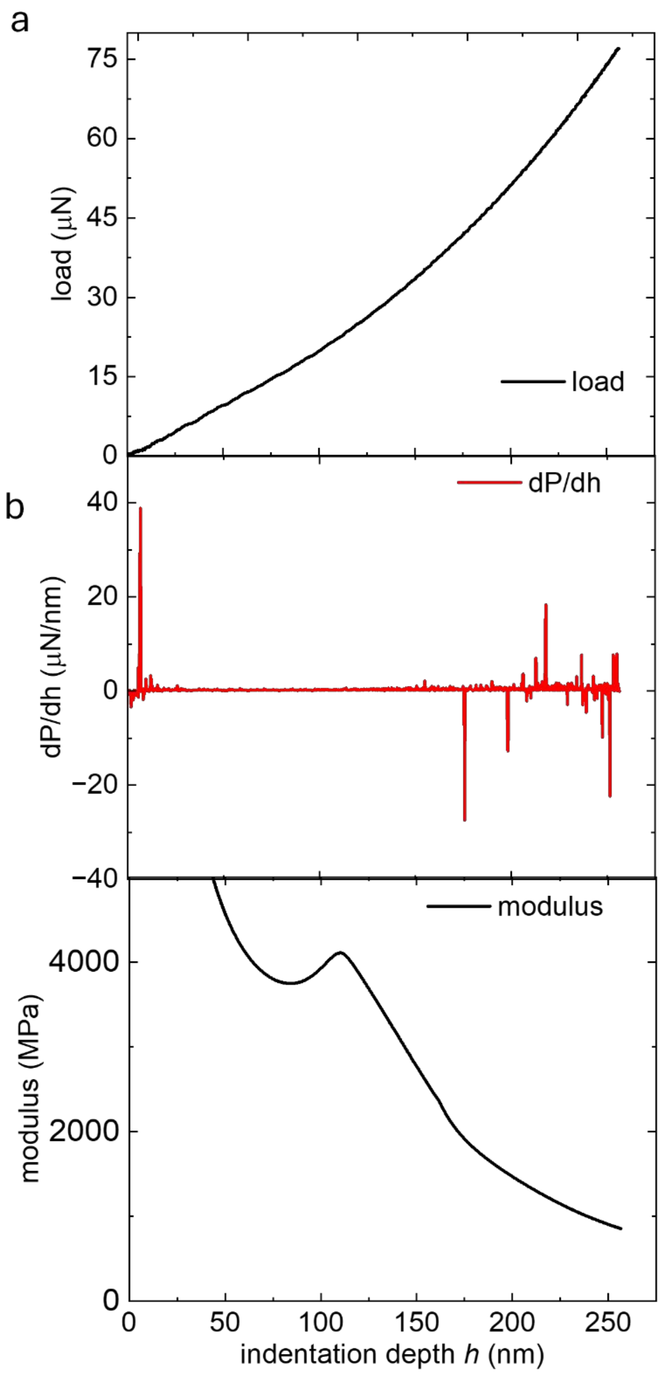

Figure S4. Representative creep analysis of a P3HT film (thickness $d \approx 4$ μm; loading rate = 16 μN s^{-1}; P_{max} = 80 μN). a) P (black line) under constant load rate at a given indentation depth. b) dP/dh (red line) at a given time of P3HT film. Later, the modulus was calculated based on dh/dP using equation 5. c) Modulus of the P3HT film as function of h.

Table S2. Values reported for the Poisson's ratio ν_f of regioregular P3HT.

ν_f	reference
0.35	[20]
0.30	[21]
0.35	[17]
0.35	[22]
0.35	[8]
0.35	[7]
0.35	[23]
0.35	[6]
0.33	[12]
0.3	[15]

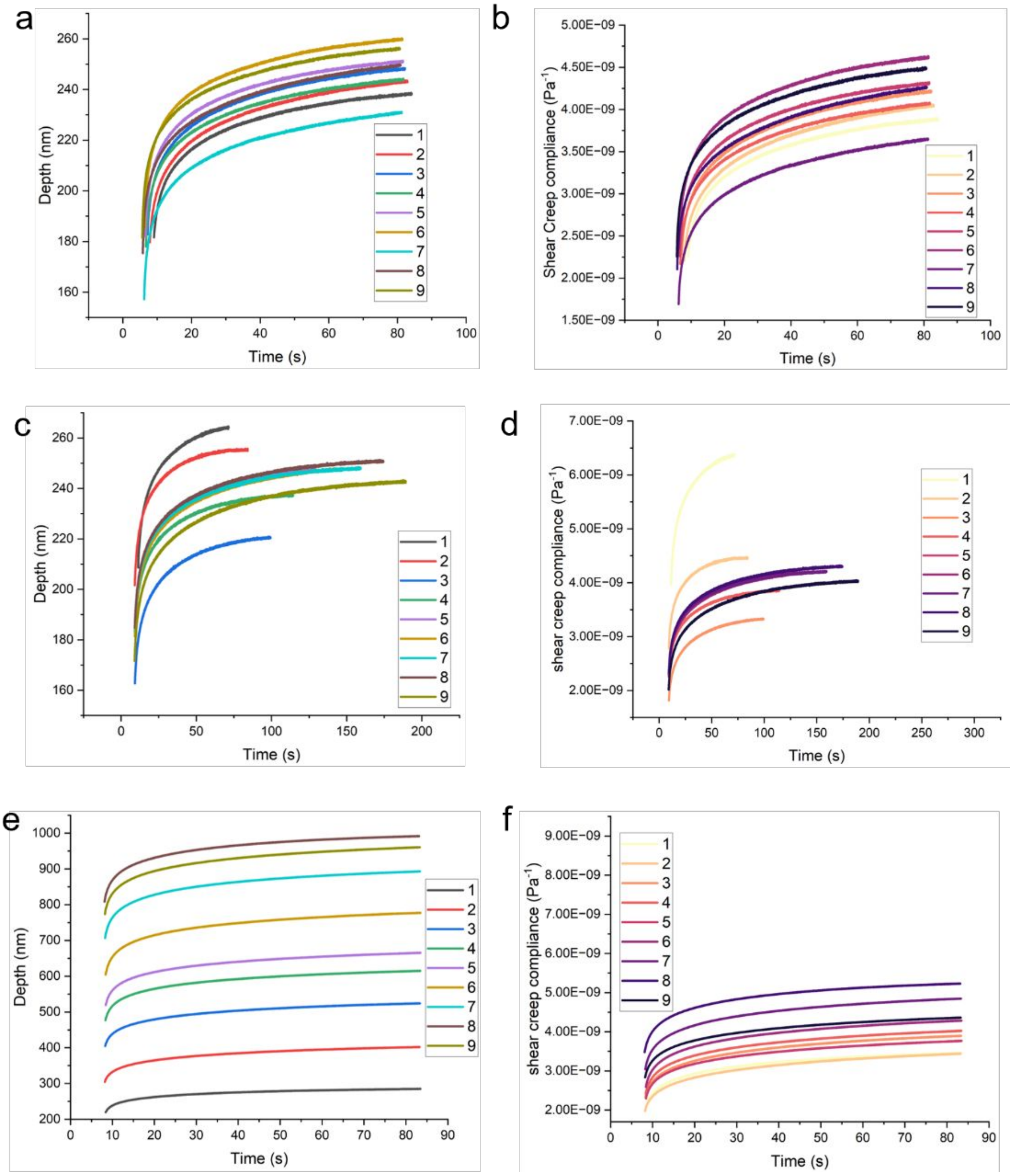

Figure S5. $h(t)$ (a, c, e) and $J(t)$ (b, d, f) of P3HT as a function of time under constant load with (a, b) the loading/unloading rate varying from dP/dt = 20 to 854 μN s^{-1} while keeping the same maximum load P_{max} = 80 μN and hold time Δt_{hold} = 75 s; (c, d) Δt_{hold} varying from 142 to 942 s while keeping the same dP/dt = 20 μN s^{-1} and P_{max} = 80 μN; and (e, f) P_{max} varying from 60 to 1500 μN while keeping the same dP/dt = 20 μN s^{-1} and Δt_{hold} = 75 s.

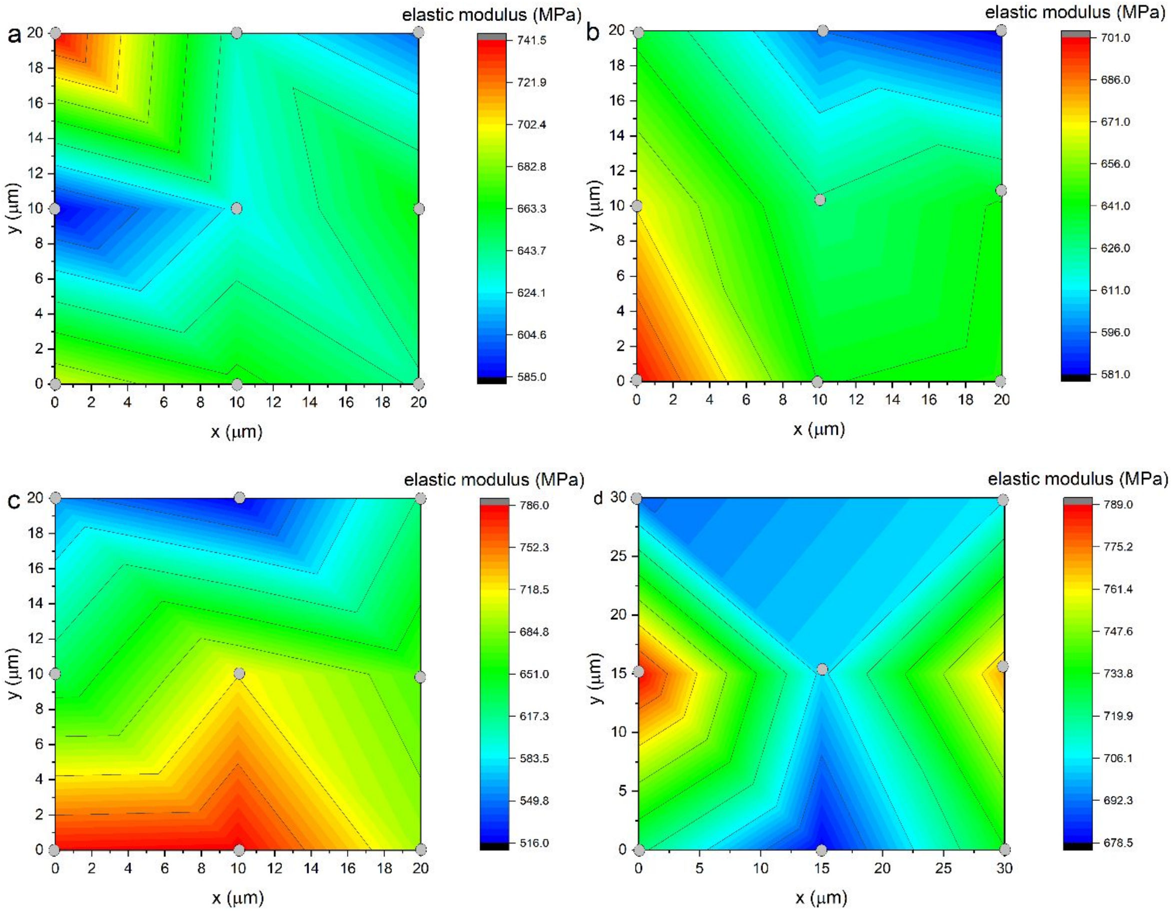

Figure S6. Contour plot of elastic modulus from creep analysis of a ≈ 4 µm thick P3HT film as a function of (a) loading/unloading rate dP/dt, (b) hold time Δt_{hold}, (c) maximum load P_{hold} and (d) when maintaining all three parameters constant.

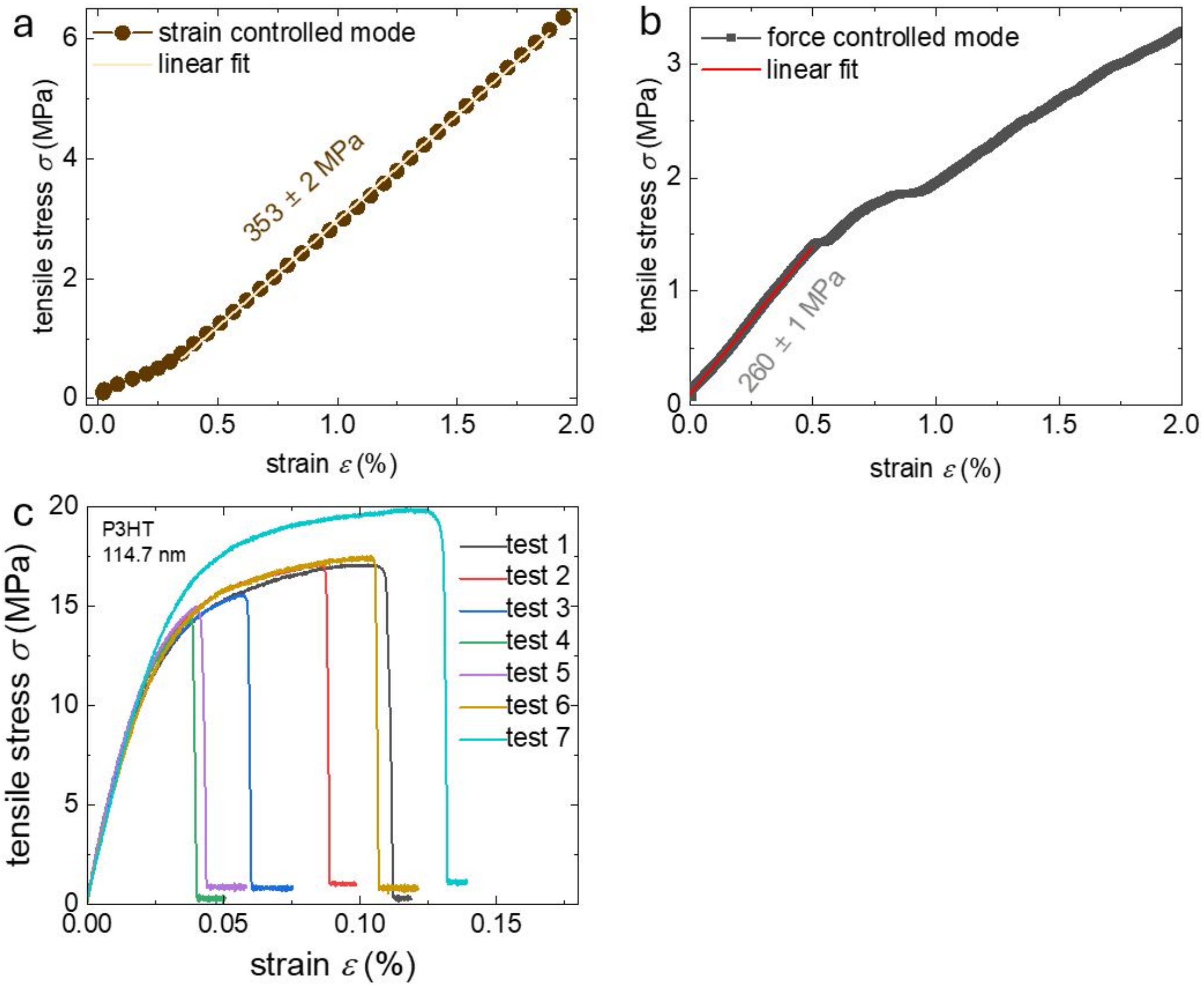

Figure S7. Tensile test of free-standing P3HT films measured in (a) strain- and (b) force-controlled mode; and (c) tensile testing of films on water (FoW).

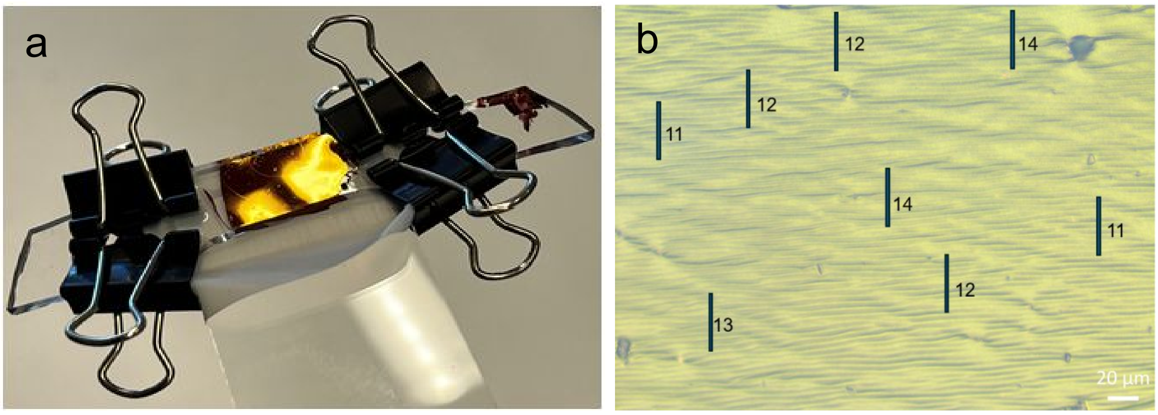

Figure S8. (a) P3HT film on a pre-strained PDMS substrate (4 %, strained from about 30.0 to 31.2 mm length) and (b) optical micrograph of the surface of a buckled P3HT film; the numbers next to each 40 micro-meter long contour line indicate the number of ridges/valleys. The strain rate was estimated as 24-36 mm min^{-1}, calculated baed on a 1.2 mm PDMS strain that was released in a few seconds.

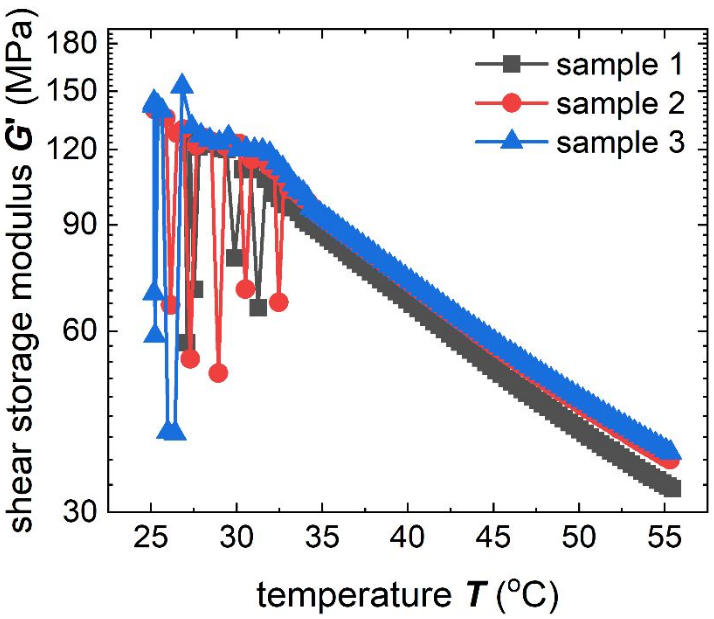

Figure S9. Shear storage modulus G' from oscillatory shear rheometry (OSR) of three P3HT disks at 1 Hz.

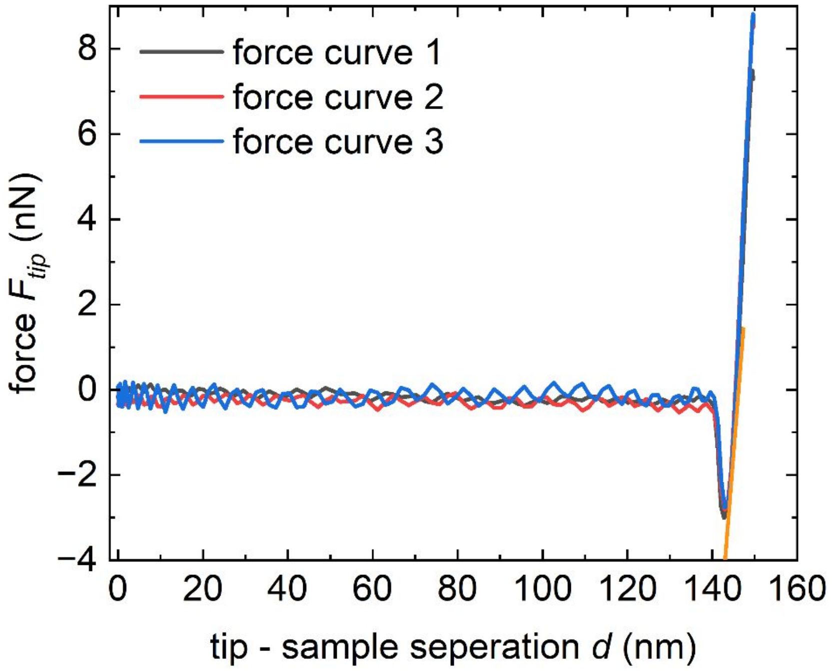

Figure S10. Force curve of P3HT film using QNM-AFM at 2000 Hz.

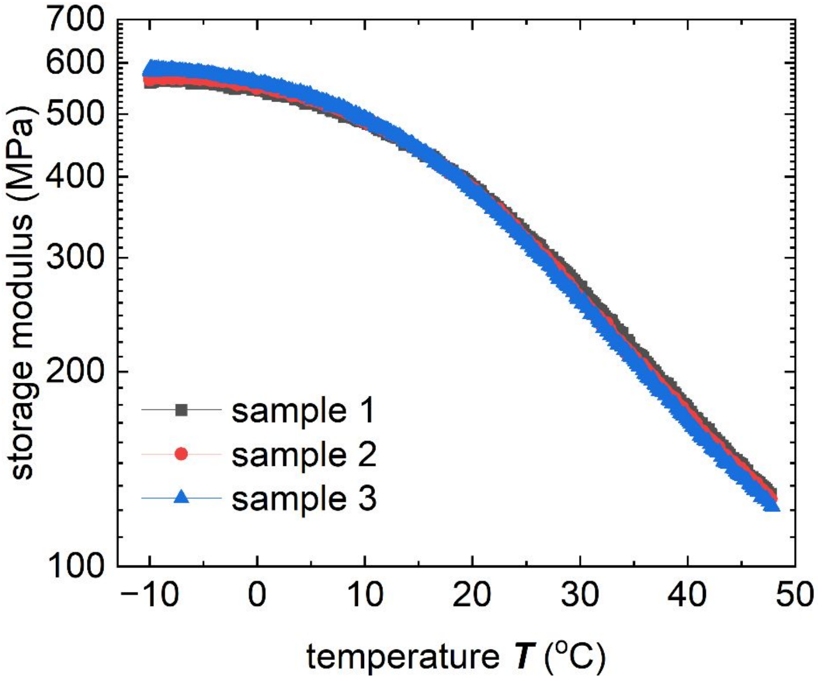

Figure S11. DMTA thermograms of free-standing P3HT films.

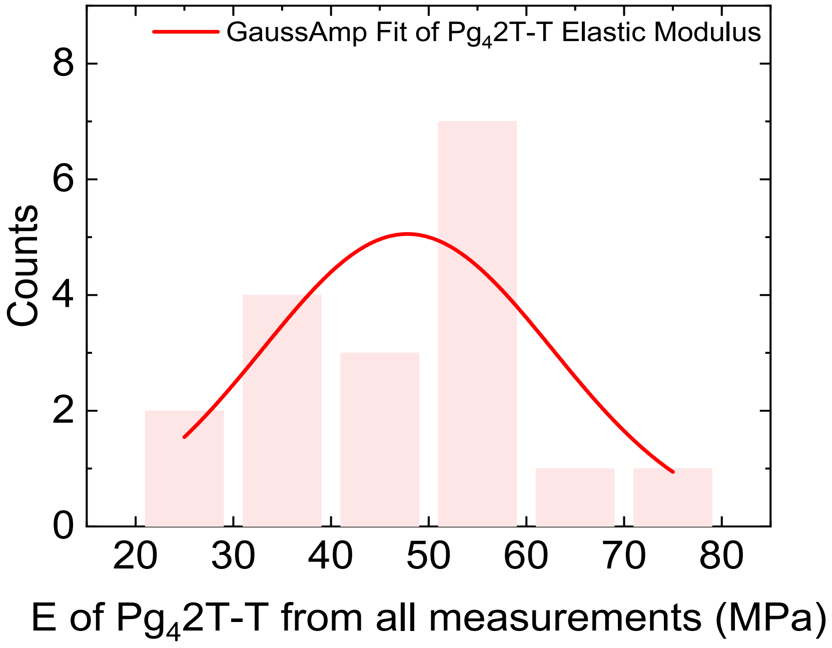

Figure S12. Gaussian fit of the frequency of all *E* values measured for p($g_4$2T-T).

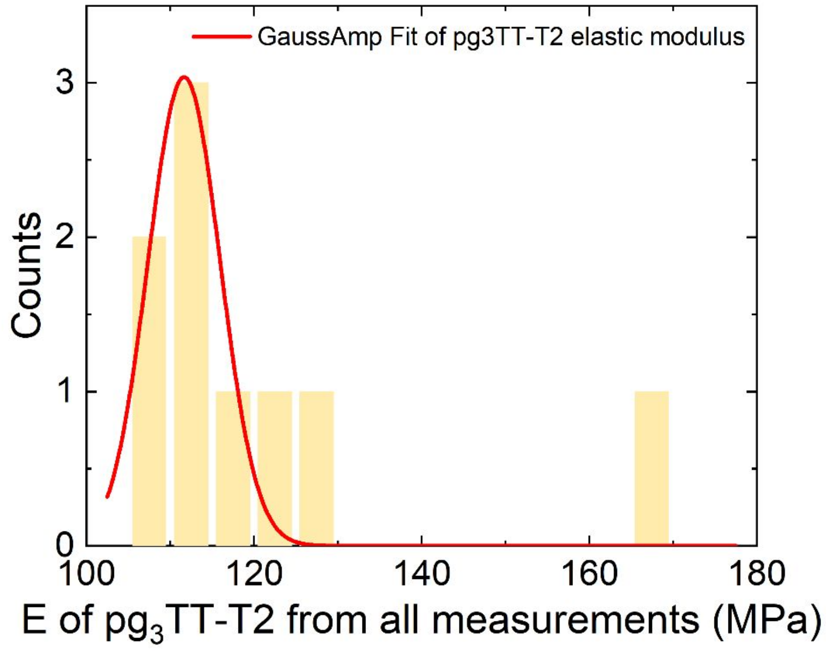

Figure S13. Gaussian fit of the frequency of all *E* values measured for p(g_3TT-T2).

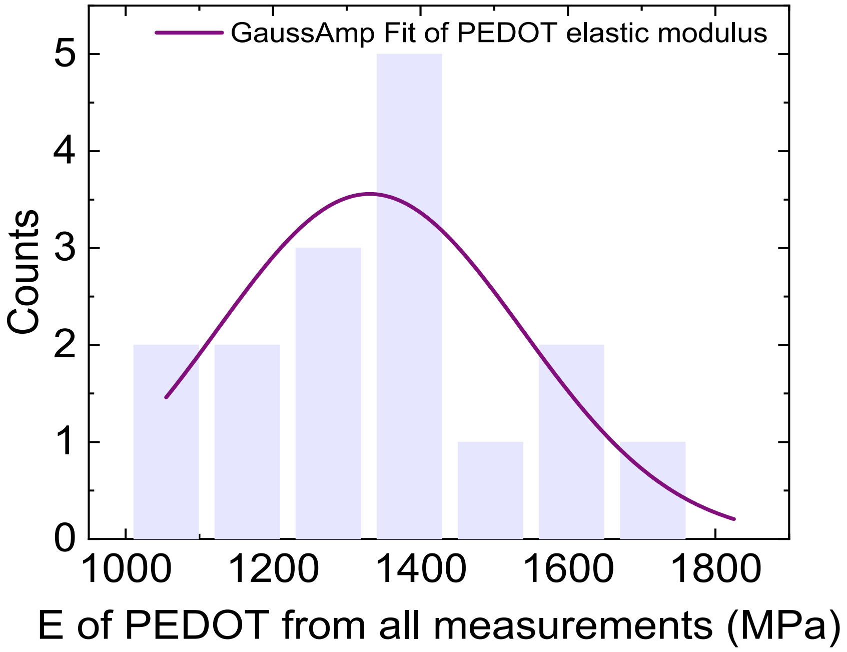

Figure S14. Gaussian fit of the frequency of all E values measured for PEDOT:PSS**.**

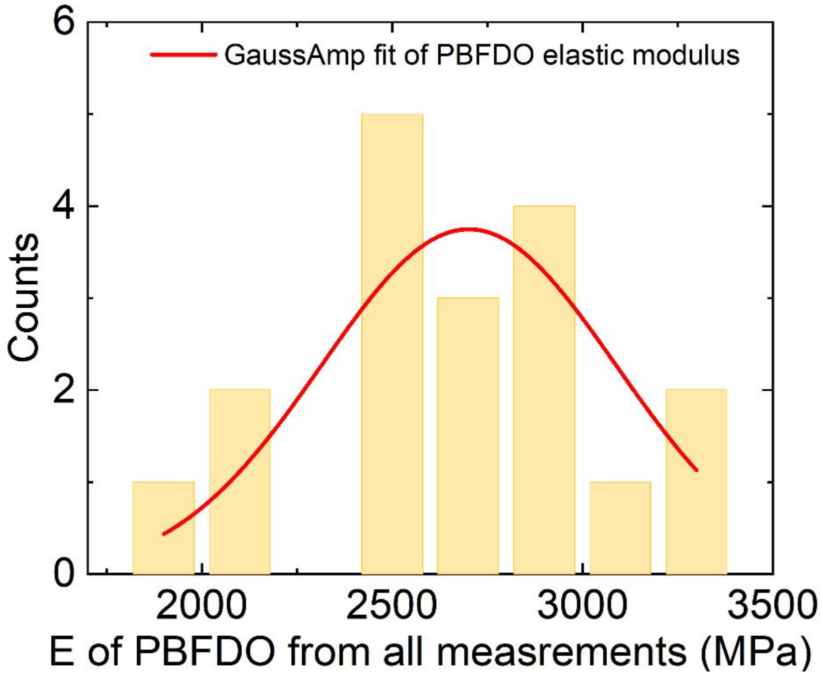

Figure S15. Gaussian fit of the frequency of all E values measured for PBFDO**.**

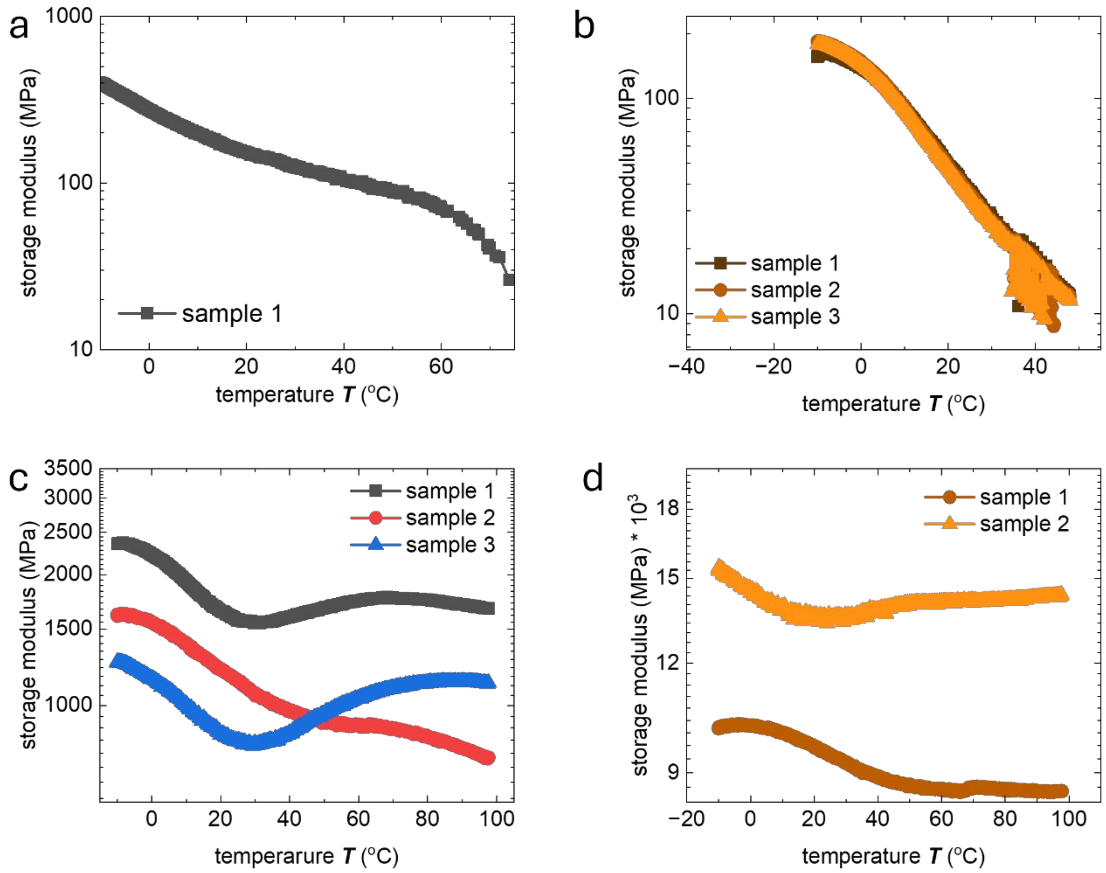

Figure S16. DMTA thermograms of free-standing films of (a) p(g_3TT-T2), (b) p($g_4$2T-T), (c) PEDOT:PSS and (d) PBFDO.

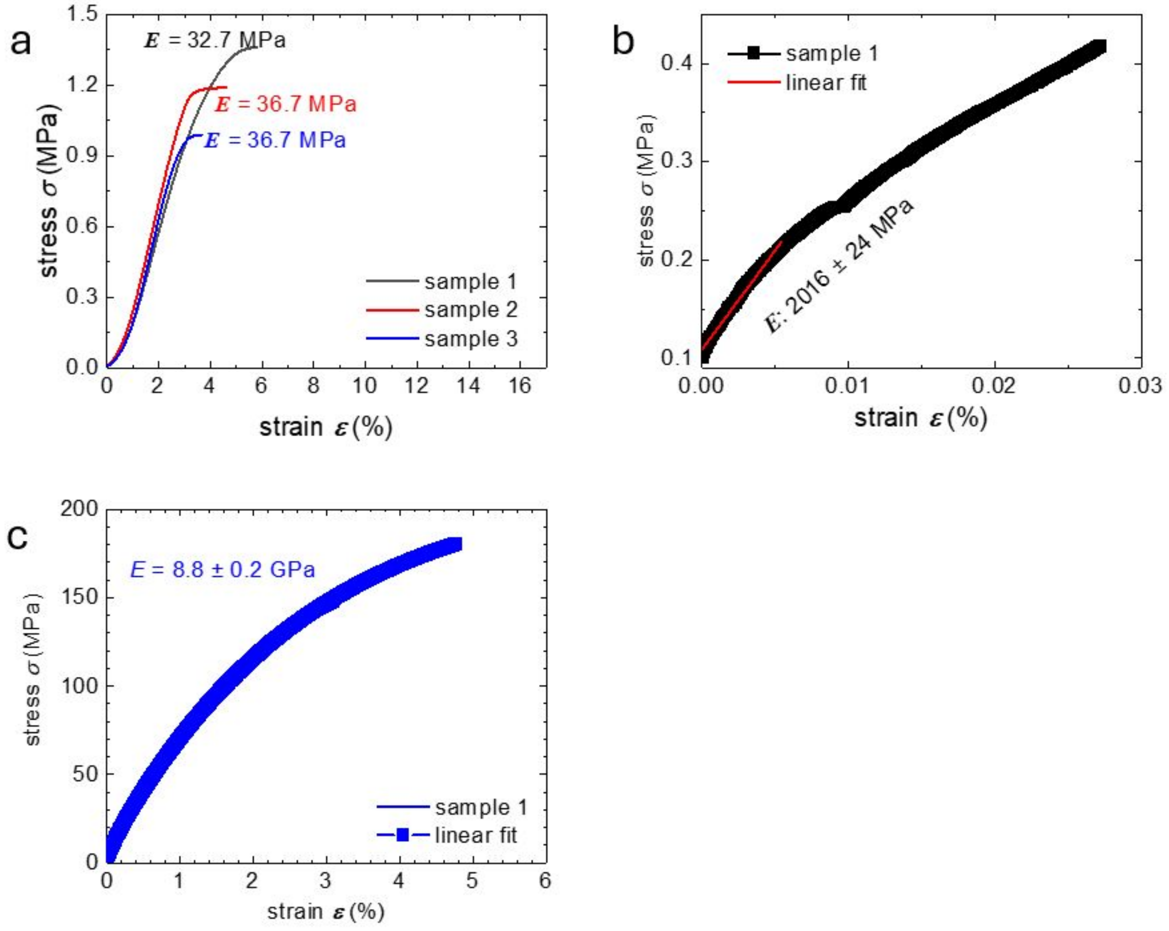

Figure S17. Tensile testing of free-standing films of (a) p(g_3TT-T2), (b) PEDOT:PSS and (c) PBFDO.

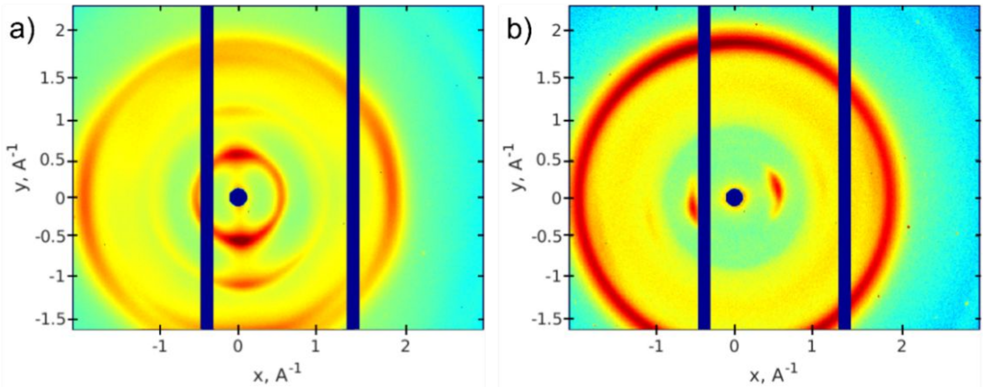

Figure S18. Transmission WAXS patterns of PBFDO films cast at 40 °C with (a) the surface and (b) the edge of the film facing the incoming beam; diffraction patterns feature arcs at q_{100} = 0.57 Å^{-1}, q_{200} = 1.14 Å^{-1} and q_{300} 1.72 Å^{-1} as well as an arc at q_{010} = 1.85 Å^{-1} in the orthogonal direction on top of a diffraction ring.

Table S3. E of p($g_4$2T-T) determined with nanoindentation (Oliver-Pharr or creep analysis), and DMTA. The tensile testing and DMTA samples were prepared by hot pressing at 170 ^{o}C, whereas nanoindentation samples were prepared by bar coating. Loading/force rate or frequency, the sample dimensions (width W, length L, thickness d), the load direction (parallel ∥ and perpendicular ⊥ to the surface of the polymer film) and the number of measurements or samples n are given. [a]Note that in case of nanoindentation deformation comprises both an out-of-plane and in-plane component.

p($g_4$2T-T)	loading rate/ frequency	$W \times L \times d$(mm × mm × µm)	∥ / ⊥	E (MPa)	n
nanoindentation (Oliver-Pharr)	20 μN s^{-1}	25× 25 × 3	⊥ [a]	245 ± 43	9
nanoindentation (creep analysis)	20 – 176 μN s^{-1}	25 × 25 × 3	⊥ [a]	47 ± 5	27
DMTA	1 Hz	6.1 × 1.4 × 64	∥	48.1 ± 0.72	3

Table S4. E of p(g_3TT-T2) determined with nanoindentation (Oliver-Pharr or creep analysis), static tensile testing (force-controlled mode) and DMTA. The tensile testing and DMTA samples were prepared by hot pressing at 170 °C, whereas nanoindentation samples are prepared by bar coating. Loading/force rate or frequency, the sample dimensions (width W, length L, thickness d), the load direction (parallel ‖ and perpendicular ⊥ to the surface of the polymer film) and the number of measurements or samples n are given. [a]Note that in case of nanoindentation deformation comprises both an out-of-plane and in-plane component.

**p(g_3TT-T2)**	**loading/force rate or frequency**	$W \times L \times d$**(mm × mm × µm)**	‖ / ⊥	E (MPa)	n
nanoindentation (Oliver-Pharr)	20 μN s^{-1}	25 × 25 × 4	⊥ [a]	689 ± 143	9
nanoindentation (creep analysis)	20-176 μN s^{-1}	25 × 25 × 4	⊥ [a]	112 ± 5	27
Static tensile testing (force controlled)	5 mN min^{-1}	13 × 7 × 80-90	‖	35 ± 2	3
DMTA	1 Hz	4 × 1 × 91	‖	153 ± 0	1

Table S5. E of PEDOT:PSS determined with nanoindentation (Oliver-Pharr or creep analysis), static tensile testing (force-controlled mode) and DMTA. The samples were drop casted on glass substrates at room temperature. Loading/force rate or frequency, the sample dimensions (width W, length L, thickness d), the load direction (parallel $\parallel$ and perpendicular $\perp$ to the surface of the polymer film) and the number of measurements or samples n are given. [a]Note that in case of nanoindentation deformation comprises both an out-of-plane and in-plane component.

PEDOT:PSS	loading/force rate or frequency	$W \times L \times d$ (mm × mm × µm)	$\parallel$ / $\perp$	E (MPa)	n
nanoindentation (Oliver-Pharr)	20 µN s^{-1}	25 × 25 × 5	$\perp$ [a]	2669 ± 68	9
nanoindentation (creep analysis)	20 – 176 µN s^{-1}	25 × 25 × 5	$\perp$ [a]	1340 ± 28	2<br>7
Static tensile testing (force controlled)	10 µN min^{-1}	8 × 2 × 5	$\parallel$	2016 ± 24	1
DMTA	1 Hz	6 × 2 × 5	$\parallel$	1427 ± 218	3

Table S6. E of PBFDO determined with nanoindentation (Oliver-Pharr or creep analysis), static tensile testing (force-controlled mode) and DMTA. The samples were drop casted on glass substrates at room temperature. Loading/force rate or frequency, the sample dimensions (width W, length L, thickness d), the load direction (parallel ‖ and perpendicular ⊥ to the surface of the polymer film) and the number of measurements or samples n are given. [a]Note that in case of nanoindentation deformation comprises both an out-of-plane and in-plane component.

**PBFDO**	**loading/force rate or frequency**	$W \times L \times d$**(mm × mm × µm)**	**‖ / ⊥**	E (MPa)	n
nanoindentation (Oliver-Pharr)	20 μN s^{-1}	25 × 25 × 14	⊥ [a]	3361 ± 82	9
nanoindentation (creep analysis)	20 – 176 μN s^{-1}	25 × 25 × 14	⊥ [a]	2680 ± 115	27
Static tensile testing (force controlled)	160 μN s^{-1}	3 × 10 × 14	‖	8800 ± 200	3
DMTA	1 Hz	7 × 20 × 13	‖	13379 ± 150	2

References

(1) Xie, R.; Lee, Y.; Aplan, M. P.; Caggiano, N. J.; Müller, C.; Colby, R. H.; Gomez, E. D. Glass Transition Temperature of Conjugated Polymers by Oscillatory Shear Rheometry. *Macromol.* **2017**, *50* (13), 5146-5154. DOI: 10.1021/acs.macromol.7b00712.

(2) Kuila, B. K.; Nandi, A. K. Structural Hierarchy in Melt-Processed Poly(3-hexyl thiophene)−Montmorillonite Clay Nanocomposites: Novel Physical, Mechanical, Optical, and Conductivity Properties. *J. Phys. Chem. B* **2006**, *110* (4), 1621-1631. DOI: 10.1021/jp055234p.

(3) Hynynen, J.; Järsvall, E.; Kroon, R.; Zhang, Y.; Barlow, S.; Marder, S. R.; Kemerink, M.; Lund, A.; Müller, C. Enhanced Thermoelectric Power Factor of Tensile Drawn Poly(3-hexylthiophene). *ACS Macro Lett.* **2019**, *8* (1), 70-76. DOI: 10.1021/acsmacrolett.8b00820.

(4) Koch, F. P. V.; Rivnay, J.; Foster, S.; Müller, C.; Downing, J. M.; Buchaca-Domingo, E.; Westacott, P.; Yu, L.; Yuan, M.; Baklar, M.; Fei, Z.; Luscombe, C.; McLachlan, M. A.; Heeney, M.; Rumbles, G.; Silva, C.; Salleo, A.; Nelson, J.; Smith, P.; Stingelin, N. The impact of molecular weight on microstructure and charge transport in semicrystalline polymer semiconductors–poly(3-hexylthiophene), a model study. *Prog. Polym. Sci.* **2013**, *38* (12), 1978-1989. DOI: 10.1016/j.progpolymsci.2013.07.009.

(5) Awartani, O.; Lemanski, B. I.; Ro, H. W.; Richter, L. J.; DeLongchamp, D. M.; O'Connor, B. T. Correlating Stiffness, Ductility, and Morphology of Polymer:Fullerene Films for Solar Cell Applications. *Adv. Energy Mater.* **2013**, *3* (3), 399-406. DOI: 10.1002/aenm.201200595.

(6) O'Connor, B.; Chan, E. P.; Chan, C.; Conrad, B. R.; Richter, L. J.; Kline, R. J.; Heeney, M.; McCulloch, I.; Soles, C. L.; DeLongchamp, D. M. Correlations between Mechanical and Electrical Properties of Polythiophenes. *ACS Nano* **2010**, *4* (12), 7538-7544. DOI: 10.1021/nn1018768.

(7) Savagatrup, S.; Makaram, A. S.; Burke, D. J.; Lipomi, D. J. Mechanical Properties of Conjugated Polymers and Polymer-Fullerene Composites as a Function of Molecular Structure. *Adv. Funct. Mater.* **2014**, *24* (8), 1169-1181. DOI: 10.1002/adfm.201302646.

(8) Tahk, D.; Lee, H. H.; Khang, D.-Y. Elastic Moduli of Organic Electronic Materials by the Buckling Method. *Macromol.* **2009**, *42* (18), 7079-7083. DOI: 10.1021/ma900137k.

(9) Zhang, S.; Ocheje, M. U.; Luo, S.; Ehlenberg, D.; Appleby, B.; Weller, D.; Zhou, D.; Rondeau-Gagné, S.; Gu, X. Probing the Viscoelastic Property of Pseudo Free-Standing Conjugated Polymeric Thin Films. *Macromol. Rapid Commun.* **2018**, *39* (14), 1800092. DOI: 10.1002/marc.201800092.

(10) Kim, J.-S.; Kim, J.-H.; Lee, W.; Yu, H.; Kim, H. J.; Song, I.; Shin, M.; Oh, J. H.; Jeong, U.; Kim, T.-S.; Kim, B. J. Tuning Mechanical and Optoelectrical Properties of Poly(3-hexylthiophene) through Systematic Regioregularity Control. *Macromol.* **2015**, *48* (13), 4339-4346. DOI: 10.1021/acs.macromol.5b00524.

(11) Rodriquez, D.; Kim, J.-H.; Root, S. E.; Fei, Z.; Boufflet, P.; Heeney, M.; Kim, T.-S.; Lipomi, D. J. Comparison of Methods for Determining the Mechanical Properties of Semiconducting Polymer Films for Stretchable Electronics. *ACS Appl. Mater. Interfaces* **2017**, *9* (10), 8855-8862. DOI: 10.1021/acsami.6b16115.

(12) Song, R.; Schrickx, H.; Balar, N.; Siddika, S.; Sheikh, N.; O'Connor, B. T. Unveiling the Stress–Strain Behavior of Conjugated Polymer Thin Films for Stretchable Device Applications. *Macromol.* **2020**, *53* (6), 1988-1997. DOI: 10.1021/acs.macromol.9b02573.

(13) Degousée, T.; Untilova, V.; Vijayakumar, V.; Xu, X.; Sun, Y.; Palma, M.; Brinkmann, M.; Biniek, L.; Fenwick, O. High thermal conductivity states and enhanced figure of merit in aligned polymer thermoelectric materials. *J. Mater. Chem. A* **2021**, *9* (29), 16065-16075. DOI: 10.1039/D1TA03377H.

(14) Li, H.-C.; Koteswara Rao, K.; Jeng, J.-Y.; Hsiao, Y.-J.; Guo, T.-F.; Jeng, Y.-R.; Wen, T.-C. Nano-scale mechanical properties of polymer/fullerene bulk hetero-junction films and their influence on photovoltaic cells. *Sol. Energy Mater. Sol. Cells* **2011**, *95* (11), 2976-2980. DOI: 10.1016/j.solmat.2011.05.039.

(15) Nanayakkara, M. P. A.; Masteghin, M. G.; Basiricò, L.; Fratelli, I.; Ciavatti, A.; Kilbride, R. C.; Jenatsch, S.; Webb, T.; Richheimer, F.; Wood, S.; Castro, F. A.; Parnell, A. J.; Fraboni, B.; Jayawardena, K. D. G. I.; Silva, S. R. P. Molecular Weight Tuning of Organic Semiconductors for Curved Organic–Inorganic Hybrid X-Ray Detectors. *Adv. Sci.* **2022**, *9* (2), 2101746. DOI: 10.1002/advs.202101746.

(16) An, X.; Wang, K.; Bai, L.; Wei, C.; Xu, M.; Yu, M.; Han, Y.; Sun, N.; Sun, L.; Lin, J.; Ding, X.; Xie, L.; Zhang, Q.; Qin, T.; Huang, W. Intrinsic mechanical properties of the polymeric semiconductors. *J. Mater. Chem. C* **2020**, *8* (33), 11631-11637. DOI: 10.1039/D0TC02255A.

(17) Mefferd, B. E.; Nambiar, V. V.; Lu, H.; Stefan, M. C. Viscoelastic Characterization of Poly(3-hexylthiophene): Determination of Young's Modulus. *ACS Appl. Polym. Mater.* **2023**, *5* (8), 6318-6324. DOI: 10.1021/acsapm.3c00939.

(18) Hay, J. Measuring Substrate-Independent Young's Modulus of Thin Films. In *MEMS and Nanotechnology, Volume 4*, New York, NY, 2011//, 2011; Proulx, T., Ed.; Springer New York: pp 45-51.

(19) Sun, N.; Mao, Z.; Zhang, X.; Tkachev, S. N.; Lin, J.-F. Hot dense silica glass with ultrahigh elastic moduli. *Scientific Reports* **2022**, *12* (1), 13946. DOI: 10.1038/s41598-022-18062-6.

(20) Root, S. E.; Savagatrup, S.; Pais, C. J.; Arya, G.; Lipomi, D. J. Predicting the Mechanical Properties of Organic Semiconductors Using Coarse-Grained Molecular Dynamics Simulations. *Macromol.* **2016**, *49* (7), 2886-2894. DOI: 10.1021/acs.macromol.6b00204.

(21) Gao, L.; Tian, Y.-m.; Hou, W.; Yan, W.-Q.; Zhong, G.-y. Piezoresistance of Poly(3-Hexylthiophene) Film. *Adv. Polym. Technol.* **2018**, *37* (3), 662-667. DOI: 10.1002/adv.21707.

(22) Menichetti, G.; Colle, R.; Grosso, G. Strain Modulation of Band Offsets at the PCBM/P3HT Heterointerface. *The Journal of Physical Chemistry C* **2017**, *121* (25), 13707-13716. DOI: 10.1021/acs.jpcc.7b02717.

(23) Savagatrup, S.; Printz, A. D.; Rodriquez, D.; Lipomi, D. J. Best of Both Worlds: Conjugated Polymers Exhibiting Good Photovoltaic Behavior and High Tensile Elasticity. *Macromol.* **2014**, *47* (6), 1981-1992. DOI: 10.1021/ma500286d.
